# Anti-Austerity Activity of Thai Medicinal Plants: Chemical Constituents and Anti-Pancreatic Cancer Activities of *Kaempferia parviflora*

**DOI:** 10.3390/plants10020229

**Published:** 2021-01-25

**Authors:** Sijia Sun, Min Jo Kim, Dya Fita Dibwe, Ashraf M. Omar, Sirivan Athikomkulchai, Ampai Phrutivorapongkul, Takuya Okada, Kiyoshi Tsuge, Naoki Toyooka, Suresh Awale

**Affiliations:** 1 Natural Drug Discovery Laboratory, Institute of Natural Medicine, University of Toyama, 2630 Sugitani, Toyama 930-0194, Japan; sunsijia4869@yahoo.com (S.S.); kiki10304@gmail.com (M.J.K.); eddydibwe@gmail.com (D.F.D.); Ashraf.omar88@gmail.com (A.M.O.); 2 Faculty of Pharmacy, Srinakharinwirot University, Nakhon Nayok 26120, Thailand; sirivan@g.swu.ac.th; 3 Faculty of Pharmacy, Chiang Mai University, Chiang Mai 50200, Thailand; amprx126@gmail.com; 4 Faculty of Engineering, University of Toyama, 3190 Gofuku, Toyama 930-8555, Japan; tokada@eng.u-toyama.ac.jp (T.O.); tsuge@sci.u-toyama.ac.jp (K.T.); toyooka@eng.u-toyama.ac.jp (N.T.); 5 Graduate School of Innovative Life Science, University of Toyama, 3190 Gofuku, Toyama 930-8555, Japan

**Keywords:** anti-austerity, preferential cytotoxicity, pancreatic cancer, *Kaempferia parviflora*

## Abstract

Human pancreatic tumor cells have an intrinsic ability to tolerate nutrition starvation and survive in the hypovascular tumor microenvironment, the phenomenon termed as “austerity”. Searching for an agent that inhibits such tolerance to nutrient starvation and kills the pancreatic cancer cells preferentially in nutrient-starvation is a unique anti-austerity strategy in anti-cancer drug discovery. In this strategy, plant extracts and compounds are tested against PANC-1 human pancreatic cancer cell line under the conditions of nutrient-deprived medium (NDM) and nutrient-rich medium (DMEM), to discover the compounds that show selective cytotoxicity in NDM. Screening of twenty-five Thai indigenous medicinal plant extracts for their anti-austerity activity against the PANC-1 human pancreatic cancer cell line in nutrient deprived medium (NDM) resulted in the identification of four active plants, *Derris scandens*, *Boesenbergia pandurata*, *Citrus hystrix*, and *Kaempferia parviflora*, with PC_50_ values 0.5–8.9 µg/mL. *K. parviflora* extract also inhibited PANC-1 cancer cell colony formation. Phytochemical investigation of *K. parviflora* extract led to the isolation of fourteen compounds, including two polyoxygenated cyclohexanes (**1** and **2**), eleven flavonoids (**3**–**13**), and *β*-sitosterol (**14**). Stereochemical assignment of compound **1** was confirmed through X-ray analysis. All isolated compounds were tested for their preferential cytotoxicity against PANC-1 cells. Among them, 5-hydroxy-7-methoxyflavone (**3**) displayed the most potent activity with a PC_50_ value of 0.8 µM. Mechanistically, it was found to induce apoptosis in PANC-1 cell death in NDM as evident by caspase cleavage. It was also found to inhibit PANC-1 cancer cell colony formation in DMEM. Therefore, compound **3** can be considered as a potential lead compound for the anticancer drug development based on the anti-austerity strategy.

## 1. Introduction

Pancreatic cancer is the deadliest cancer type with a five-year survival rate less than 5%. During 2019, 35,700 deaths occurred, which made pancreatic cancer occupy the third and fourth primary cause of cancer-related deaths among females and males, respectively [1]. The aggressiveness and high mortality rate of pancreatic cancer are further complicated by the poor diagnosis of that disease during the initial phase [2]. Moreover, most pancreatic cancer patients, at the time of diagnosis, already developed metastasized lesions, making surgery almost impossible. Furthermore, pancreatic tumors show intrinsic resistance to chemotherapeutic agents in clinical practice [3]. Therefore, there is an urgent need for finding alternative agents to combat this disease.

Pancreatic tumors are highly aggressive. However, the microenvironment of pancreatic tumors is inherently hypovascular in nature, leading to a poor nutrient supply to aggressively proliferating tumor cells. Pancreatic tumor cells adapt within such nutrient deficient microenvironment by altering their energy metabolism to tolerate the extreme nutrient starvation condition, a phenomenon termed as “austerity” in cancer biology [4]. The search for agents that can inhibit cancer cells’ tolerance to nutrition starvation, displaying preferential cytotoxic activity against cancer cells under the nutrient-deprived condition without toxicity under normal-nutrient condition, is a powerful anti-austerity approach in anticancer drug discovery [5]. Previous studies utilizing this strategy resulted in the discovery of several potent anti-austerity agents from traditionally used medicinal plants, including those from Japan [6], Southeast Asia [7,8], and Congo [9]. In our continuous effort to discover new anti-austerity agents, we screened twenty-five selected Thai indigenous medicinal plants extracts for their anti-austerity activity against the PANC-1 human pancreatic cancer cell line. Among them, the EtOH extracts of *Derris scandens* (PC_50_ = 0.8 µg/mL), *Boesenbergia pandurata* (PC_50_ = 0.5 µg/mL), and *Citrus hystrix* (PC_50_ = 8.9 µg/mL) and the CH_2_Cl_2_ extract of *Kaempferia parviflora* (PC_50_ = 3.3 µg/mL) were found to display the most potent activities. Furthermore, the CH_2_Cl_2_ extract of *Kaempferia parviflora* (Zingiberaceae) rhizomes was found to inhibit PANC-1 colony formation in the nutrient-rich condition.

*K. parviflora* (black ginger) is a species with deep purple-colored rhizomes. It has been reported to exhibit antimalarial, antiviral, antimycobacterial, and anti-ulcer activities [10,11]. It also exhibited antitumor activity against Hela human cervical and SKOV3 ovarian cancer cells [12,13]. However, there are no prior studies on the anticancer activities of *K. parviflora* against pancreatic cancer cells. Therefore, this active extract was subjected to a phytochemical investigation study which resulted in isolation of fourteen secondary metabolites, including two polyoxygenated cyclohexanes (**1** and **2**), eleven flavonoids (**3**–**13**) and *β*-sitosterol (**14**). We herein report the screening results of twenty-five plants extracts against PANC-1 pancreatic cancer cell line in nutrient-deprived condition; the effect of the active extracts on PANC-1 cell morphology, migration, and colony formation; and the phytochemical investigation of *K. parviflora* as well as the anti-austerity activity of its constituents.

## 2. Results and Discussion

### 2.1. Anti-Austerity Activity of Thai Medicinal Plants

Herbal medicines are the only affordable treatment options in many developing countries. In Thailand, indigenous medicinal plants and condiments are traditionally used to treat many diseases, including cancer [14]. In this study, the extracts of twenty-five selected Thai indigenous medicinal plants and condiments were tested against PANC-1 human pancreatic cancer cell line for its preferential cytotoxic activity (anti-austerity activity) in nutrient-deprived medium (NDM) and standard nutrient-rich medium (DMEM). The results are presented as preferential cytotoxicity (PC_50_) values, which represent the concentration that causes 50% cancer cell death in NDM (Table 1). Among the tested plants, four extracts exhibited potent activities. These include the EtOH extracts of *Derris scandens* (PC_50_ = 0.8 µg/mL) and *Citrus hystrix* (PC_50_ = 8.9 µg/mL) and the CH_2_Cl_2_ extracts of *Boesenbergia pandurata* (PC_50_ = 0.5 µg/mL) and *Kaempferia parviflora* (PC_50_ = 3.3 µg/mL). 

Among the active extracts, the phytochemical investigation on *C. hystrix* has been carried out previously, and bergamottin was identified as its active principle having the ability to inhibit PANC-1 cell migration and colony formation, as well as inhibit the Akt/mTOR signaling pathway [15]. In this study, we carried out phytochemical investigation of the next active extract, *K. parviflora* and the anti-austerity activities of its constituents.

### 2.2. Assessment of PANC-1 Cell Death Induced by K. parviflora Extract

**Figure 1:**
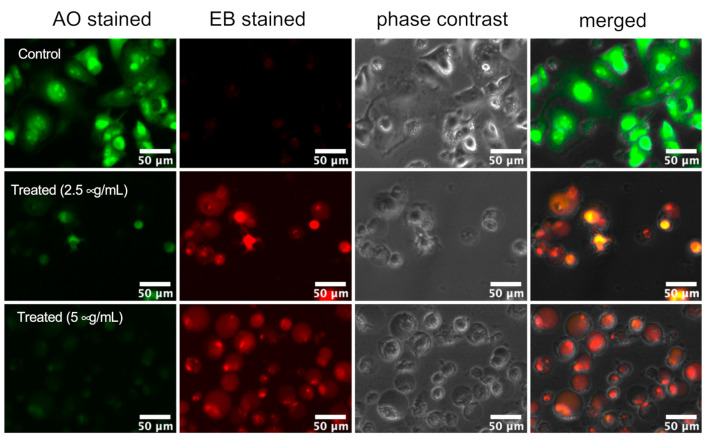
Morphological changes of PANC-1 cells induced by *K. parviflora* extract in nutrient-deprived medium (NDM). PANC-1 tumor cells were treated with *K. parviflora* extract at the indicated concentrations in NDM in a 12-well plate and incubated for 24 h. Cells were stained with ethidium bromide (EB) and acridine orange (AO) and photographed under fluorescence (red and green) and phase-contrast modes using an EVOS FL digital microscope (Thermo Fisher Scientific, Carlsbad, CA, USA) (20× objective).

The hypovascular nature of pancreatic tumors causes the limited access of the rapidly proliferating cancer cells to essential nutrients. Therefore, these cells are under constant metabolic stress. To survive under such extreme “austerity” conditions, the cancer cells alter their energy metabolism and tolerate the nutrient starvation condition. Human pancreatic cancer cells such as PANC-1 have been shown to survive for prolong periods of over 72 h, even in complete nutrient deprivation medium. In this study, *K. parviflora* extract was tested for its anti-austerity activity against PANC-1 cancer cells cultured in nutrient-deprived medium (NDM) and normal nutrient-rich medium (DMEM). Since the extract showed potent anti-austerity activity (PC_50_ = 3.3 µg/mL), it was further studied for its effect against PANC-1 cell morphology using ethidium bromide–acridine orange (EB-AO) double staining assay [9]. AO is a cell membrane permeable dye which emits bright-green fluorescence upon entering living cells, while EB can permeate only cells undergoing apoptosis or necrosis emitting predominant red fluorescence [9]. In this study, PANC-1 cells treated with the *K. parviflora* extract (2.5 and 5 µg/mL) and the untreated control were incubated for 24 h in NDM, and then stained with the EB/AO reagent. As shown in Figure 1, untreated PANC-1 cells showed intact morphology emitting bright green fluorescence, suggesting 100% cell survival even under complete nutrition starvation. However, treatment with the *K. parviflora* extract (2.5 and 5 µg/mL) led to a concentration-dependent increase in the cells emitting red fluorescence showing altered cellular morphology, indicative of dead cells (Figure 1).

### 2.3. Effect of K. parviflora Extract on PANC-1 Colony Formation

**Figure 2:**
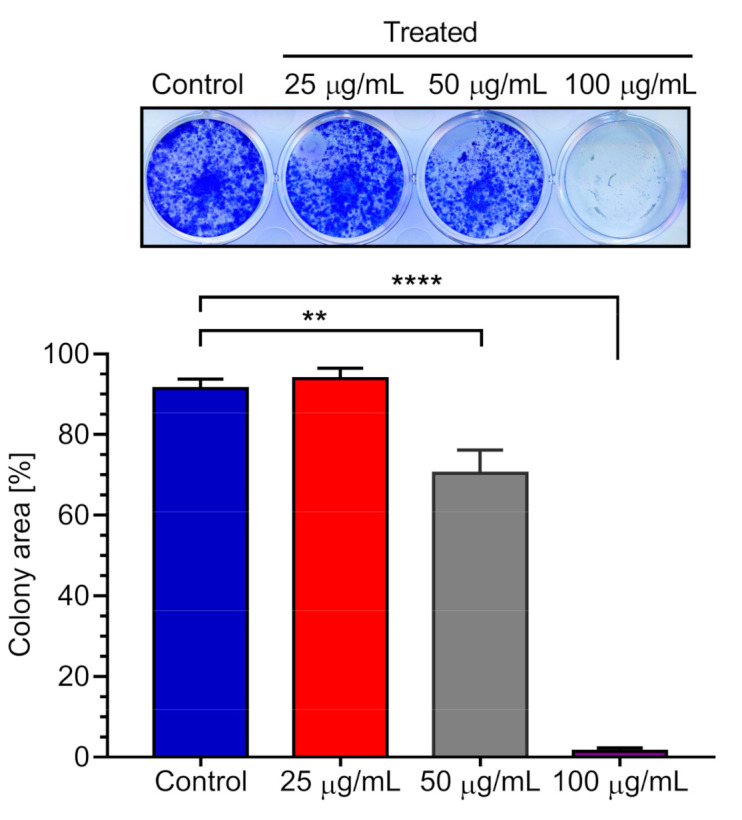
Effect of *Kaempferia parviflora* CH_2_Cl_2_ extract on colony formation by PANC-1 cells. Representative wells showing PANC-1 cell colonies. Graph showing mean values of the area occupied by PANC-1 cell colonies (three replications). Statistical analysis was carried out with students t-test (see Figure S11). **** *p* < 0.0001, ** *p* < 0.01 when compared with the untreated control group.

Pancreatic cancer cells are highly metastatic. During the metastasis process, tumor cells migrate into the distant organs, such as liver and lung, where there are sufficient vasculatures and nutrients to grow into tumor colonies by invading the normal tissues. Extracts and compounds having inhibitory activity against colony formation in nutrient-rich condition might have possible therapeutic benefits against pancreatic cancer metastasis and colony formation. The effect of *K. parviflora* extract for its potential to inhibit colony formation was investigated. PANC-1 cells were exposed to CH_2_Cl_2_ extract at concentrations of 25, 50, and 100 μg/mL in DMEM for 24 h. The medium was then replaced with fresh DMEM and placed in a CO_2_ incubator to allow colony formation for 12 days. Notably, treatment with *K. parviflora* extract at the concentration of 50 µg/mL significantly inhibited colony formation of PANC-1 cells with the total inhibition at 100 µg/mL (Figure 2).

### 2.4. Chemical Constituents of K. parviflora and Their Anti-Austerity Activity against the PANC-1 Human Pancreatic Cancer Cells

**Figure 3:**
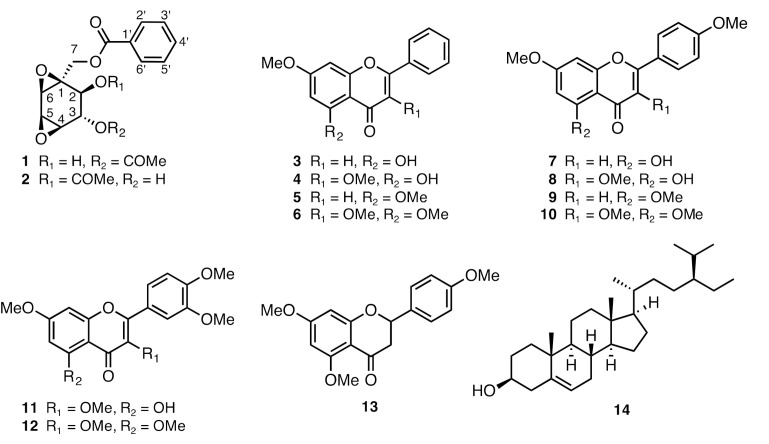
Structures of isolated compounds from *K. parviflora.*

*K. parviflora* showed potent anti-austerity activity against PANC-1 cells with PC_50_ 3.3 µg/mL, as well as colony formation inhibitory activity. Therefore, to find the active constituents responsible for the observed activities, phytochemical investigation was carried out, which resulted in the isolation of fourteen secondary metabolites, including two polyoxygenated cyclohexanes (**1** and **2**) and eleven flavonoids (**3**-**13**). The isolated compounds were identified using NMR spectroscopic analysis as (−)-4-benzoyloxymethyl-3,8-dioxatricyclo[5.1.0.0^2,4^]octane-5,6-diol 6-acetate (**1**) [16], (+)-4-benzoyloxymethyl-3,8-dioxatricyclo[5.1.0.0^2,4^]octane-5,6-diol 5-acetate (**2**) [16], 5-hydroxy-7-methoxyflavone (**3**) [17], 5-hydroxy-3,7-dimethoxyflavone (**4**) [17], 5,7-dimethoxyflavone (**5**) [17], 3,5,7-trimethoxyflavone (**6**) [17], 5-hydroxy-7,4′-methoxyflavone (**7**) [17], 5-hydroxy-3,7,4′-methoxyflavone (**8**) [17], 5,7,4′-trimethoxyflavone (**9**) [17], 3,5,7,4′-tetramethoxyflavone (**10**) [17], 5-hydroxy-3,7,3′,4′-tetramethoxyflavone (**11**) [17], 3,5,7,3′,4′-pentamethoxyflavone (**12**) [17], 5,7,4′-trimethoxyflavonone (**13**) [18], and *β*-sitosterol (**14**) [19] (Figure 3).

**Figure 4:**
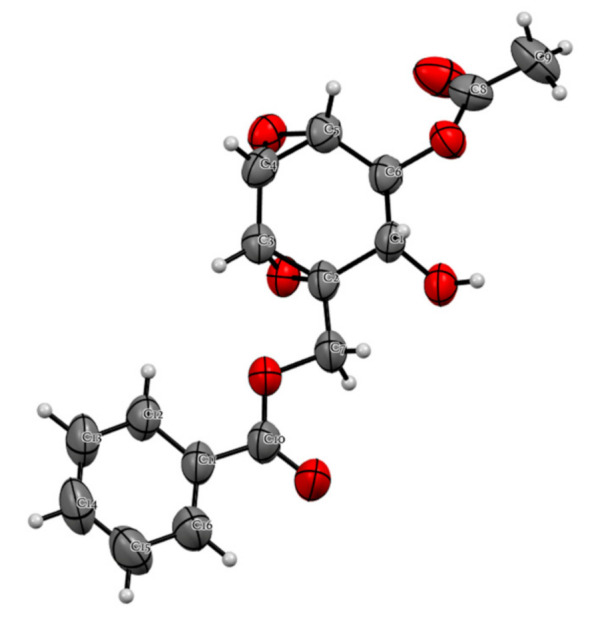
Anisotropic displacement ellipsoid plot of C_16_H_16_O_7_ at the 70% probability level; hydrogen atoms are drawn as spheres of arbitrary radius.

Among the isolated compounds, the absolute configurational assignment of the polyoxygenated cyclohexane type compounds, such as **1**, remains a challenging task. NMR spectroscopy and ECD calculations are often used for the absolute configuration assignment of these compounds. However, there are cases of incorrect assignments that require structural revisions [20]. Until now, the absolute configuration of **1** remains unclear [16]. X-ray crystallography is one of the decisive tools for the absolute configuration and structural elucidation of natural compounds. In the present study, compound **1** was crystallized and subjected to further X-ray analysis which confirmed its absolute configuration (see Table 2 and Figure 4, Figures S1–S8).

**Figure 5:**
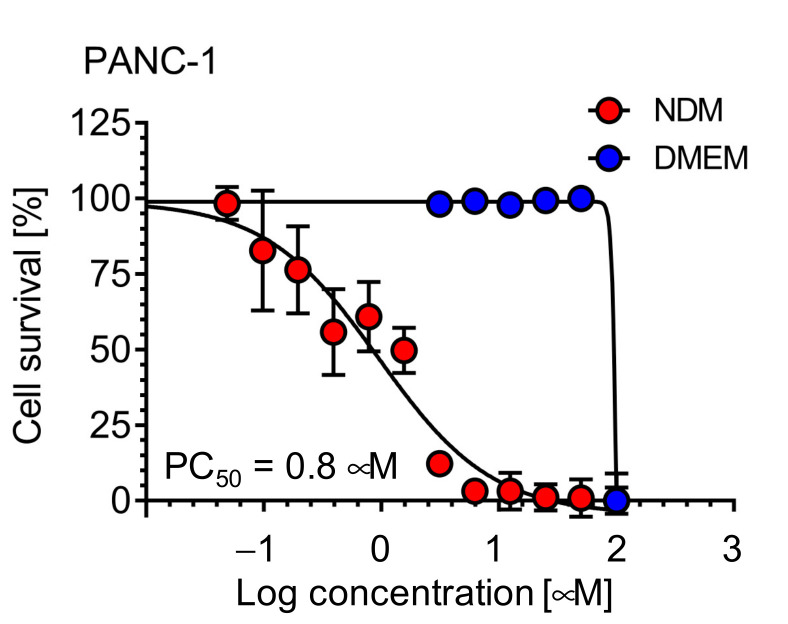
Preferential cytotoxic activity (anti-austerity activity) of 5-hydroxy-7-methoxyflavone (**3**) against the PANC-1 human pancreatic cancer cell line in nutrient-deprived medium (NDM) and Dulbecco’s modified Eagle’s medium (DMEM).

All compounds (**1**–**14**) were tested for anti-austerity activity against PANC-1 human pancreatic cancer cell lines in standard nutrient-rich (DMEM) and nutrient-deprived (NDM) conditions adopting the “anti-austerity” strategy. This strategy targets the ability of pancreatic cancer cells to survive severe nutrient deprivation without affecting normal cells growing under normal nutrient condition. Among the tested compounds, 5-hydroxy-7-methoxyflavone (**3**) showed the most potent anti-austerity activity in NDM with a PC_50_ value of 0.8 µM (eqv. 0.2 µg/mL), which is stronger than the extract having PC_50_ 3.3 µg/mL (Table 2 and Figure 5).

Upon inspection of the structures and observed activities of isolated compounds, a clear SAR relationship was observed. The flavonoids (**3**–**13**) in general displayed stronger activity compared to isolated polyoxygenated cyclohexanes (**1** and **2**) and *β*-sitosterol (**14**). Among flavonoids, compounds **3**, **5**, and **6** without OMe group at B-ring were found to be stronger than corresponding methoxylated counterparts (**7**−**12**). Similarly, flavonoids without OMe group at C-ring displayed stronger activities (**3** > **4**, **5** > **6**, **9** > **10**). Therefore, absence of OMe groups at B- and C-ring in flavone skeleton is essential for enhancement of activity (see Table 3 and Figures S9 and S10).

### 2.5. Investigation of the Mechanism of Cell Death Induced by 5-Hydroxy-7-Methoxyflavone (***3***) in NDM

**Figure 6:**
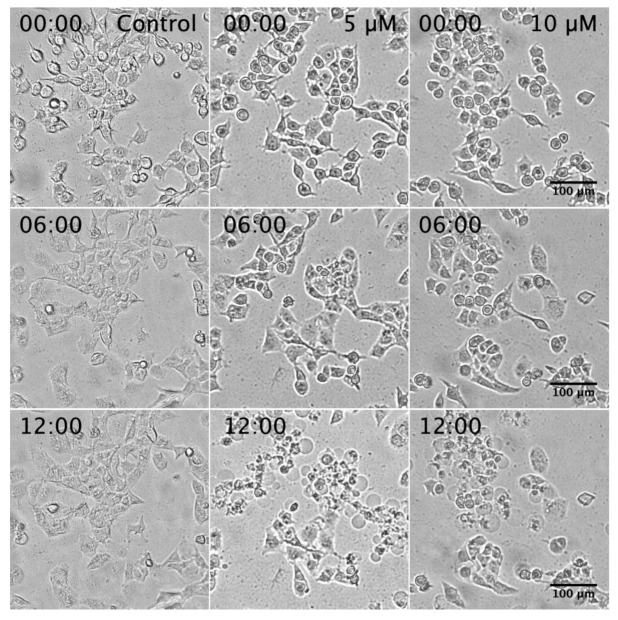
Captures of the real-time effect of 5-hydroxy-7-methoxyflavone (**3**) at 5 and 10 μM on PANC-1 cells in NDM. A live-cell imaging system was used to capture images were every 15 min during 24 h. (Please insert supplementary Video S1 at this section in the web version of this article).

To capture the real-time effects of 5-hydroxy-7-methoxyflavone (**3**) on PANC-1 cells, a time-lapse live imaging experiment was carried out. The control and PANC-1 cells treated with **3** (5 and 10 µM) in NDM were incubated for 24 h within a CO_2_ incubator equipped with a digital CytoSMART live cell imaging system (Lonza, Basel, Switzerland). Ninety-seven images were captured at an interval of 15 min for 24 h. As shown in Figure 6 and real-time Video S1, PANC-1 cells treated with **3** at 5 and 10 µM showed cell shrinkage and rounded morphology after 8 h of treatment, followed by plasma membrane blebbing after 11 h, leading to total cell death within 24 h. 

**Figure 7:**
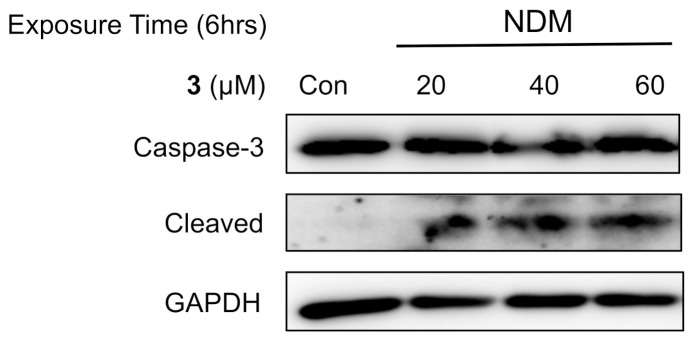
Effect of 5-hydroxy-7-methoxyflavone (**3**) against the expression of caspase-3 under NDM in PANC-1 cells.

To further investigate whether cell death induced by 5-hydroxy-7-methoxyflavone (**3**) involved apoptosis in NDM, a Western blot analysis was carried out. Caspase-3 is activated by proteolytic cleavage during apoptosis [21]. Thus, the induction of apoptosis can be followed by an increase of the cleaved-caspase 3. Treatment of PANC-1 cells with **3** for a short time of 6 h in NDM showed a significant increment in cleaved caspase-3 expression in a concentration-dependent manner (Figure 7), suggesting the induction of apoptosis by compound **3**.

### 2.6. Effect of 5-Hydroxy-7-methoxyflavone (**3**) on PANC-1 Colony Formation in DMEM

**Figure 8:**
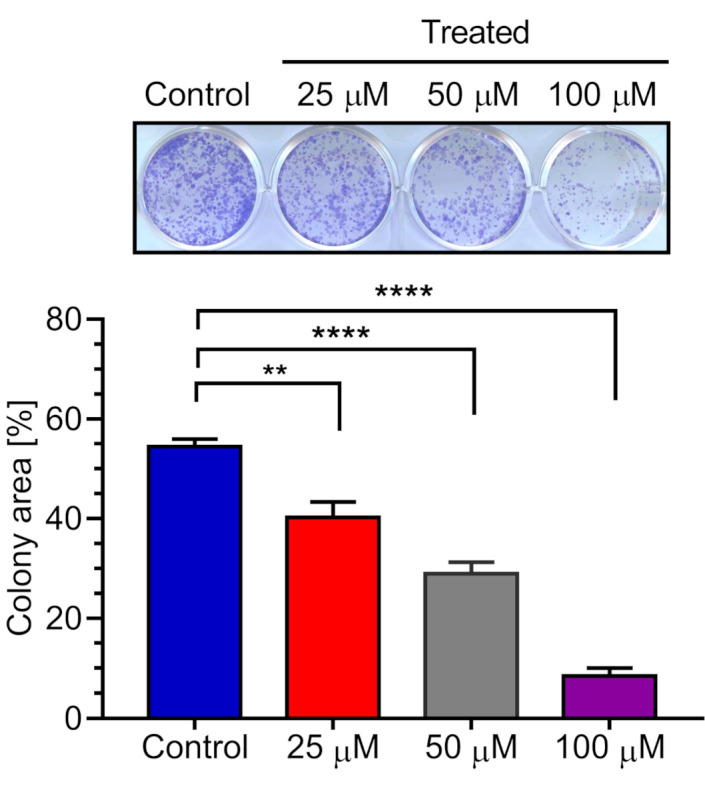
Effect of 5-hydroxy-7-methoxyflavone (**3**) on colony formation by PANC-1 cells. Representative wells showing PANC-1 cell colonies. Graph showing mean values of the area occupied by PANC-1 cell colonies (three replications). Statistical analysis was carried out with students *t*-test (see Figure S12). **** *p* < 0.0001, ** *p* < 0.01 when compared with the untreated control group.

During cancer metastasis, the invading cancer cells adapt to foreign tissue microenvironments forming small colonies of cancer cells, which then grow into large tumors [22]. Most pancreatic tumor patients quickly develop liver metastases soon after the time of diagnosis [23]. Thus, 5-hydroxy-7-methoxyflavone (**3**) was further investigated for its effect on colony formation in DMEM. PANC-1 cells were exposed to **3** at concentrations of 25, 50, and 100 µM in nutrient-rich DMEM medium for 24 h. The medium was then replaced with fresh DMEM and placed in a CO_2_ incubator to allow colony formation for 12 days. Contrary to this, treatment with **3** showed a significant concentration-dependent inhibition on the colony formation in DMEM (Figure 8). It should be noted here that compound **3** showed preferential cytotoxic activity in nutrient-deprived condition without apparent toxicity in nutrient-rich DMEM medium. Therefore, non-cytotoxic concentrations of **3** in DMEM are used in the colony formation assay. Since metastasized colonies at distant organs such as liver have sufficient nutrient to proliferate, colony formation assay in DMEM at the non-toxic concentration reveals its potential to inhibit cancer at distant organs without causing apparent toxicity to the organ itself, thus preventing possible severe side effects, which otherwise are observed with the conventional chemotherapeutic agents in clinical practice.

## 3. Materials and Methods

### 3.1. General Experimental Procedures

^1^H (400 MHz) and ^13^C (100 MHz) NMR spectra were recorded using JEOL ECX400 (Tokyo, Japan) Delta spectrometer with TMS as an internal standard, and chemical shifts are expressed in *δ* values in parts per million (ppm), and coupling constants (*J*) are given in hertz (Hz). Multiplicities are denoted as singlet (s), doublet (d), doublet of doublets (dd), triplet (t), or multiplet (m). A Büchi MPLC C-605 (Flawil, Switzerland) binary gradient pump system was used to perform medium-pressure liquid chromatography (MPLC) with normal-phase silica gel (silica gel 60N, spherical, neutral, 40–50 μm, Kanto Chemical). Pre-coated silica gel 60F_254_ and RP-18F_254_ plates (Merck, 0.25 or 0.50 mm thickness) were used for analytical and preparative TLC. 

### 3.2. Plant Material

Twenty-five plants parts were purchased from an organic farmer’s market at Muang district, Chiang Mai province, Thailand in June 2016: *Phlogacanthus pulcherrimus* (leaves), *Clinacanthus nutans* (leaves), *Polyscias fruticosa* (shoot), *Eupatorium stoechadosmum* (leaves), *Gymnema inodorum* (leaves), *Dolichandrone serrulata* (flowers), *Plukenetia volubilis* (endocarps), *Plectranthus amboinicus* (leaves), *Hibiscus sabdariffa* (calyx), *Derris scandens* (flowers), *Antidesma thwaitesianum* (leaves), *Antidesma thwaitesianum* (fruits), *Piper sarmentosum* (leaves), *Boesenbergia pandurata* (rhizomes), *Polygonum odoratum* (leaves, twigs), *Breynia vitis-idaea* (leaves), *Morinda citrifolia* (fruits), *Murraya paniculate* (leaves), *Clausena anisata* (leaves), *Citrus hystrix* (leaves), *Citrus hystrix* (fruit peels), *Zanthoxylum myriacanthum* (pericarps), *Zanthoxylum myriacanthum* (leaves), *Houttuynia cordata* (leaves), and *Kaempferia parviflora* (rhizomes). The specimens were identified by Dr. Wannaree Charoensup, Botanist, Department of Pharmaceutical Sciences, Faculty of Pharmacy, Chiang Mai University. Voucher specimens have been deposited at the Plant Herbarium Museum, Faculty of Pharmacy, Chiang Mai University, Thailand.

### 3.3. Phytochemical Investigation of K. parviflora

Dried rhizomes of *K. parviflora* were ground to a fine powder (962 g), which were then extracted with CH_2_Cl_2_ under sonification (2 L, 90 min, × 3). The CH_2_Cl_2_ solution was filtered and evaporated under reduced pressure to obtain the CH_2_Cl_2_ extract (33.0 g, PC_50_ 3.82 μg/mL). This extract was chromatographed on silica gel by MPLC using *n*-hexane–EtOAc–MeOH solvent system to give five fractions [fr. 1, 1.0 g; fr. 2, 4.6 g; fr. 3, 1.1 g; fr. 4, 5.8 g; fr. 5, 15.0 g]. Fraction 1 was chromatographed by normal-phase MPLC using an *n*-hexane–EtOAc gradient system to afford three subfractions [fr. 1-1, 106 mg; fr. 1-2, 436 mg; fr. 1-3, 421 mg]. Subfraction 1-3 was recrystallized from EtOAc to yield 5-hydroxy-3,7-dimethoxyflavone (4, 280 mg). Fraction 2 was subjected to normal-phase MPLC with an *n*-hexane–EtOAc gradient system to afford three subfractions [fr. 2-1, 1.4 g; fr. 2-2, 2.1 g, fr. 2-3, 1.0 g]. Subfraction 2-1 was further purified by normal-phase MPLC using an *n*-hexane/EtOAc gradient system to give 5-hydroxy-7-methoxyflavone (3, 644 mg) and *β*-sitosterol (14, 62 mg). Subfraction 2-2 was recrystallized from EtOAc to yield 5-hydroxy-3,7,4’-methoxyflavone (8, 314 mg). Subfraction 2-3 was separated by normal-phase MPLC with an *n*-hexane/EtOAc gradient system to give 5-hydroxy-7,4’-methoxyflavone (7, 261 mg) and 5-hydroxy-3,7,3’,4’-tetramethoxyflavone (11, 245 mg). Fraction 3 was rechromatographed by normal-phase MPLC using an *n*-hexane–EtOAc gradient system yielding two subfractions [fr. 3-1, 594 mg; fr. 3-2, 509 mg]. Subfraction 3-1 was separated by normal-phase MPLC with an *n*-hexane/EtOAc gradient system to give (−)-4-benzoyloxymethyl-3,8-dioxatri-cyclo[5.1.0.0^2,4^]octane-5,6-diol 6-acetate (1, 5.1 mg), (+)-4-benzoyloxymethyl-3,8-dioxatri-cyclo[5.1.0.0^2,4^]octane-5,6-diol 5-acetate (2, 4.6 mg) and 5,7,4’-trimethoxyflavonone (13, 9.9 mg). Fraction 4 was subjected to normal-phase MPLC with an EtOAc–MeOH gradient system to afford three subfractions [fr. 4-1, 249 mg; fr. 4-2, 33.9 mg, fr. 4-3, 5.5 g]. Subfractions 4-1 and 4-2 were recrystallized from EtOAc to yield 3,5,7-trimethoxyflavone (6, 249 mg). Subfraction 4-3 was separated by normal-phase MPLC with an *n*-hexane/EtOAc gradient system to give 5,7-dimethoxyflavone (5, 8.8 mg) and 3,5,7,4’-tetramethoxyflavone (10, 629 mg). Fraction 5 was subjected to normal-phase MPLC with an EtOAc–MeOH gradient system to afford two subfractions [fr. 5-1, 14.0 g; fr. 5-2, 1.0 g]. Subfraction 5-1 was purified by normal-phase MPLC with an EtOAc/MeOH gradient system to give 5,7,4’-trimethoxyflavone (9, 222 mg) and 3,5,7,3’,4’-pentamethoxyflavone (12, 433 mg).

### 3.4. Chemicals and Antibodies

HEPES was purchased from Dōjindo Laboratories (Kumamoto, Japan). Fetal bovine serum was purchased from Nichirei Biosciences Inc. (Tokyo, Japan). Antibiotic/antimycotic solution was purchased from Sigma–Aldrich. Nutrient-deprived medium was prepared according to a previously described protocol [6]. Rabbit polyclonal antibodies to caspase-3, cleaved caspase-3, and GAPDH were purchased from Cell Signaling Technology (Danvers, MA, USA). Horseradish peroxidase-conjugated goat polyclonal anti-rabbit and rabbit polyclonal anti-goat immunoglobulins were purchased from DakoCytomation (Glostrup, Denmark). Other chemicals were purchased from Wako (Wako Pure Chemical, Osaka, Japan).

### 3.5. Cell Line and Cell Culture

The PANC-1 (RBRC-RCB2095) human pancreatic cancer cell line was purchased from the Riken BRC cell bank. The cell line was maintained in standard DMEM with 10% FBS supplement, 0.1% sodium bicarbonate, and 1% antibiotic antimycotic solution.

### 3.6. Preferential Cytotoxicity against PANC-1 Pancreatic Cancer Cells

Preferential cytotoxicities of the twenty-five Thai indigenous medicinal plants’ extracts and the isolated compounds of *K. parviflora* were determined by a procedure described previously [6]. In brief, PANC-1 cells (1.5 × 10^4^ cells/100 μL/well) in DMEM were seeded in 96-well plates and incubated for 24 h. The cells were then washed twice with Dulbecco’s phosphate-buffered saline (PBS) and replaced with either DMEM or nutrient-deprived medium (NDM) containing serially diluted test samples and incubated for 24 h. The medium was then replaced with 100 μL of DMEM containing 10% WST-8 cell counting kit solution. After 3 h of incubation, the absorbance was measured at 450 nm. Cell viability was calculated from the mean values for three wells using the following equation:

(1) $$Cell\;viability\;\left(\%\right)=\;\frac{Abs_{\left(test\;samples\right)}\;-\;Abs_{\left(blank\right)}}{Abs_{\left(control\right)}\;-\;Abs_{\left(blank\right)}}\;\times \;100\%$$

The preferential cytotoxicity was expressed as the concentration at which 50% of cells died preferentially in NDM (PC_50_).

### 3.7. Morphological Analysis

PANC-1 cells (5 × 10^4^ cells/well) were seeded in DMEM in a 12-well plate and incubated at 37 °C under humidified 5% CO_2_ for 24 h. Phosphate-buffered saline (PBS) was then used to wash the cells followed by treatment with NDM alone (the control) or NDM containing *K. parviflora* extract (2.5 and 5 μg/mL). Both the treated and control cells were incubated for 24 h and then treated with EB/AO reagent and images were captured using an Evos FL digital microscope (20× objective) with phase-contrast and fluorescence modes.

### 3.8. Colony Formation Inhibition Assay

PANC-1 cells (2000 cells/well) were plated in 12-well plates at a density of in DMEM (2 mL/well) and incubated at 37 °C under humidified 5% CO_2_ for 24 h for cell attachment. The cells were then treated with *K. parvifolia* extract or 5-hydroxy-7-methoxyflavone (3) in DMEM and were allowed to grow at 37 °C under humidified 5% CO_2_ for 12 days. Cells were then washed with PBS, fixed with 4% formaldehyde, and stained with crystal violet for 15 min. Each experiment was performed in triplicates. Colony area was measured using the ImageJ plugin “Colony Area” [24].

### 3.9. Western Blot Analysis

Western blot analysis was carried out by a procedure described previously [9]. In brief, the PANC-1 cells were treated with three different concentrations of compound 3 (20, 40, and 60 μM) for 6 h in NDM. Proteins were then separated by gel electrophoresis on a polyacrylamide gel containing 0.1% sodium dodecyl sulfate and transferred to polyvinylidene fluoride membranes. The membranes were blocked with Block Ace (DS Pharma Medical, Suita, Japan), washed with PBS containing 0.1% polyoxyethylenesorbitan monolaurate (Wako Pure Chemical, Osaka, Japan), and incubated overnight with caspase-3, cleaved caspase-3 and GAPDH antibodies (Cell Signaling Technology, Danvers, MA, USA). After washing, the membranes were incubated for 45 min at room temperature with horseradish peroxidase-conjugated anti-rabbit or anti-goat immunoglobulins as the secondary antibody. The bands were detected with an enhanced chemiluminescence solution (Bio-Rad, Hercules, CA, USA).

### 3.10. Statistical Analysis

Data are presented as means ± SD; the statistical significance was calculated by student’s *t*-test using GraphPad Prism.

## 4. Conclusions

Screening of twenty-five Thai indigenous medicinal plants extracts for anti-austerity activity against the PANC-1 human pancreatic cancer cell line in NDM resulted in the identification of four active plants, *Derris scandens*, *Boesenbergia pandurata*, *Citrus hystrix*, and *Kaempferia parviflora*, with PC_50_ values 0.5–8.9 µg/mL. *K. parviflora* extract was found to inhibit colony formation, and this was subjected to phytochemical investigation, that resulted in the isolation of fourteen compounds (**1**−**14**). The absolute configuration of **1** was achieved by X-ray crystallography. Among the isolated compounds, 5-hydroxy-7-methoxyflavone (**3**) was identified as a potential anti-austerity agent. Compound **3** was found to induce apoptosis in PANC-1 cell death in NDM, as well as inhibit PANC-1 cancer cell colony formation in DMEM. Therefore, 5-hydroxy-7-methoxyflavone (**3**) is a promising lead structure for the anticancer drug development based on the anti-austerity strategy.

## Figures and Tables

**Table 1:** Thai medicinal plants used in present study and their preference cytotoxic activity against PANC-1 cells.

Scientific Name	Part Used	Family	Extraction Solvent	Preferential Cytotoxicity ^a^
*Phlogacanthuspulcherrimus*	leaves	Acanthaceae	70% EtOH	>100
*Clinacanthus nutans*	leaves	Acanthaceae	70% EtOH	>100
*Polyscias fruticosa*	shoot	Araliaceae	70% EtOH	12.0 ± 1.5
*Eupatorium stoechadosmum*	leaves	Asteraceae	70% EtOH	>100
*Gymnema inodorum*	leaves	Asclepiadaceae	70% EtOH	38.0 ± 1.7
*Dolichandrone serrulata*	flowers	Bignoniaceae	70% EtOH	>100
*Plukenetia volubilis*	endocarps	Euphorbiaceae	70% EtOH	71.5 ± 1.5
*Plectranthus amboinicus*	leaves	Lamiaceae	70% EtOH	88.9 ± 1.8
*Hibiscus sabdariffa*	calyx	Malvaceae	70% EtOH	>100
*Derris scandens*	flowers	Papilionaceae	95% EtOH	0.8 ± 0.2
*Antidesma thwaitesianum*	leaves	Phyllanthaceae	70% EtOH	>100
*Antidesma thwaitesianum*	fruits	Phyllanthaceae	95% EtOH	90.8 ± 1.7
*Piper sarmentosum*	leaves	Piperaceae	70% EtOH	91.9 ± 1.8
*Boesenbergia pandurata*	rhizomes	Zingiberaceae	100% CH_2_Cl_2_	0.5 ± 0.1
*Polygonum odoratum*	leaves & twigs	Polgonaceae	70% EtOH	31.3 ± 1.4
*Breynia vitis-idaea*	leaves	Euphorbiaceae	70% EtOH	87.9 ± 1.3
*Morinda citrifolia*	fruits	Rubiaceae	70% EtOH	>100
*Murraya paniculata*	leaves	Rutaceae	70% EtOH	44.9 ± 1.8
*Clausena anisata*	leaves	Rutaceae	70% EtOH	>100
*Citrus hystrix*	leaves	Rutaceae	70% EtOH	56.4 ± 1.6
*Citrus hystrix*	fruit peels	Rutaceae	70% EtOH	8.9 ± 1.4
*Zanthoxylum myriacanthum*	pericarps	Rutaceae	70% EtOH	21.7 ± 2.1
*Zanthoxylum myriacanthum*	leaves	Rutaceae	70% EtOH	81.3 ± 1.8
*Houttuynia cordata*	leaves	Saururaceae	70% EtOH	>100
*Kaempferia parviflora*	rhizomes	Zingiberaceae	100% CH_2_Cl_2_	3.3 ± 0.5

^a^ Concentration (µg/mL) at which 50% of cancer cells were killed preferentially in the nutrient-deprived medium (NDM) without causing toxicity in the nutrient-rich medium (DMEM). Each value represents a mean ± SD of three replications.

**Table 2:** NMR spectroscopic data in CDCl_3_ for compound **1.**

Position	Compound 1	
	*δ*_C_, Type	*δ*_H_ (*J* in Hz)
1	56.0, C	
2	66.0, CH	4.13, dd (10.4, 4.8)
3	69.8, CH	5.17, dd (4.8, 2.8)
4	51.0, CH	3.31, t (2.8)
5	48.0, CH	3.55, t (2.8)
6	53.8, CH	3.70, d (2.8)
7a	64.8, CH_2_	4.54, d (12.0)
7b	64.8, CH_2_	4.42, d (12.0)
1’	129.4, C	
2’,6’	129.9, CH	8.05, m
3’,5’	128.7, CH	7.47, m
4’	133.7, CH	7.59, m
C=O	166.1, C	
C=O	170.0, C	
2-OH		2.69, d (10.4)
Me	20.6, CH_3_	1.89, s

**Table 3:** Anti-austerity activity (PC_50_) *^a^* of the compounds **1**−**14** against PANC-1 human pancreatic cancer cell line.

Compound	PC_50_, μM *^a^*	Compound	PC_50_, μM *^a^*
**Extract**	3.3 ± 0.5 μg/mL		
**1**	>100	**9**	50.3 ± 1.5
**2**	>100	**10**	85.3 ± 1.8
**3**	0.8 ± 0.1	**11**	>100
**4**	>100	**12**	>100
**5**	16.0 ± 1.3	**13**	63.7 ± 1.5
**6**	73.2 ± 2.2	**14**	>100
**7**	>100	Arctigenin Should *^b^*	0.8 ± 0.2
**8**	>100		

*^a^* Concentration at which 50% of cells were killed preferentially in the nutrient-deprived medium (NDM) without causing toxicity in the nutrient-rich medium (DMEM). *^b^* Positive control. Each value represents a mean ± SD of three replications.

## Supplementary Materials

The following are available online at https://www.mdpi.com/2223-7747/10/2/229/s1, Figures S1–S8: Copies of spectroscopic data for compound **1**. Figure S9: Preferential cytotoxic activity of *Kaempferia parviflora* CH_2_Cl_2_ extract and all isolated compounds against the PANC-1 in NDM and DMEM. Figure S10: Preferential cytotoxic activity (anti-austerity activity) of 5-hydroxy-7-methoxyflavone (**3**) against the PANC-1 human pancreatic cancer cell line in nutrient-deprived medium (NDM). Figure S11: Effect of *Kaempferia parviflora* CH_2_Cl_2_ extract on colony formation of PANC-1 cells. Figure S12. Effect of 5-hydroxy-7-methoxyflavone (**3**) on colony formation of PANC-1 cells. File S2: Experimental details of X-ray of compound **1**. Video S1: Movie showing the real-time effect of 5-hydroxy-7-methoxyflavone (**3**) on PANC-1 cells in NDM for 24 h.
