# Supplementary material for: Anti-Austerity Activity of Thai Medicinal Plants: Chemical Constituents and Anti-Pancreatic Cancer Activities of Kaempferia parviflora
Source: Plants (Basel). 2021 Jan 25;10(2):229. doi: 10.3390/plants10020229 (PMC7911922; doi:10.3390/plants10020229)
Supplement: Supplementary file 1 [file plants-10-00229-s001.zip › Supplementary Material 1.docx]

Supplementary Material

Anti-austerity Activity of Thai Medicinal Plants: Chemical Constituents and Anti-pancreatic Cancer Activities of *Kaempferia parviflora*

Sijia Sun^1^, Min Jo Kim^1^, Dya Fita Dibwe^1^, Ashraf M. Omar^1^, Sirivan Athikomkulchai^2^, Ampai Phrutivorapongkul^3^, Takuya Okada^4^, Kiyoshi Tsuge^4^, Naoki Toyooka^4,5^, Suresh Awale^1,*^

^1^ Natural Drug Discovery Laboratory, Institute of Natural Medicine, University of Toyama, 2630 Sugitani, Toyama 930-0194, Japan

^2^ Faculty of Pharmacy, Srinakharinwirot University, Nakhon Nayok, 26120, Thailand.

^3^ Faculty of Pharmacy, Chiang Mai University, Chiang Mai 50200, Thailand.

^4^ Faculty of Engineering, University of Toyama, 3190 Gofuku, Toyama 930-8555, Japan.

^5^ Graduate School of Innovative Life Science, University of Toyama, 3190 Gofuku, Toyama 930-8555, Japan

***** Correspondence: suresh@inm.u-toyama.ac.jp; Tel: +81-76-434-7640.

Table of Contents

Experimental details………………………………………………………………....…..1

Figure S1. ^1^H NMR spectrum of the compound **1** (400 MHz – CDCl_3_) .…………....2

Figure S2. ^13^C NMR spectrum of the compound **1** (100 MHz – CDCl_3_) …………....3

Figure S3. HMQC NMR spectrum of the compound **1** …………………………...…4

Figure S4. HMBC NMR spectrum of the compound **1** ……………...…………..…...5

Figure S5. COSY NMR spectrum of the compound **1** …………………......................6

Figure S6. IR spectrum of the compound **1** ……………..…………….…………...…..7

Figure S7. UV spectrum of the compound **1** …………………..…………....................8

Figure S8. HR-FAB-MS spectrum of the compound **1** …………...……...……………9

Figure S9. Preferential cytotoxic activity of *Kaempferia parviflora* CH_2_Cl_2_ extract and all isolated compounds against the PANC-1 in NDM and DMEM...…………….…10

Figure S10. Preferential cytotoxic activity (anti-austerity activity) of 5-hydroxy-7-methoxyflavone (**3**) against the PANC-1 human pancreatic cancer cell line in nutrient-deprived medium (NDM) …………………..…………………………..........11

**Experimental details**

Each crude sample (200 g) was cut into small pieces and extracted with 70% EtOH, 95% EtOH or CH_2_Cl_2_ under sonification (2 L, 90 min, × 3). The solution was then filtered and evaporated under reduced pressure to obtain the extracts. Each extract (~ 2 mg) was dissolved initially in DMSO, followed by dilution with the corresponding medium to give a final DMSO concentration of 0.05%−1%. The diluted solutions were examined to determine their preferential cytotoxicity against the PANC‐1 human pancreatic cancer cell line under nutrient‐deprived conditions.

**Figure S1.** ^1^H NMR spectrum of the compound **1** (400 MHz, CDCl_3_)

**Figure S2.** ^13^C NMR spectrum of the compound **1** (100 MHz, CDCl_3_)

**Figure S3.** HMQC NMR spectrum of the compound **1**

**Figure S4.** HMBC NMR spectrum of the compound **1**

**Figure S5.** COSY NMR spectrum of the compound **1**

**Figure S6.** IR spectrum of the compound **1**

**Figure S7.** UV spectrum of the compound **1**

**Figure S8.** HR-FAB-MS spectrum of the compound **1**


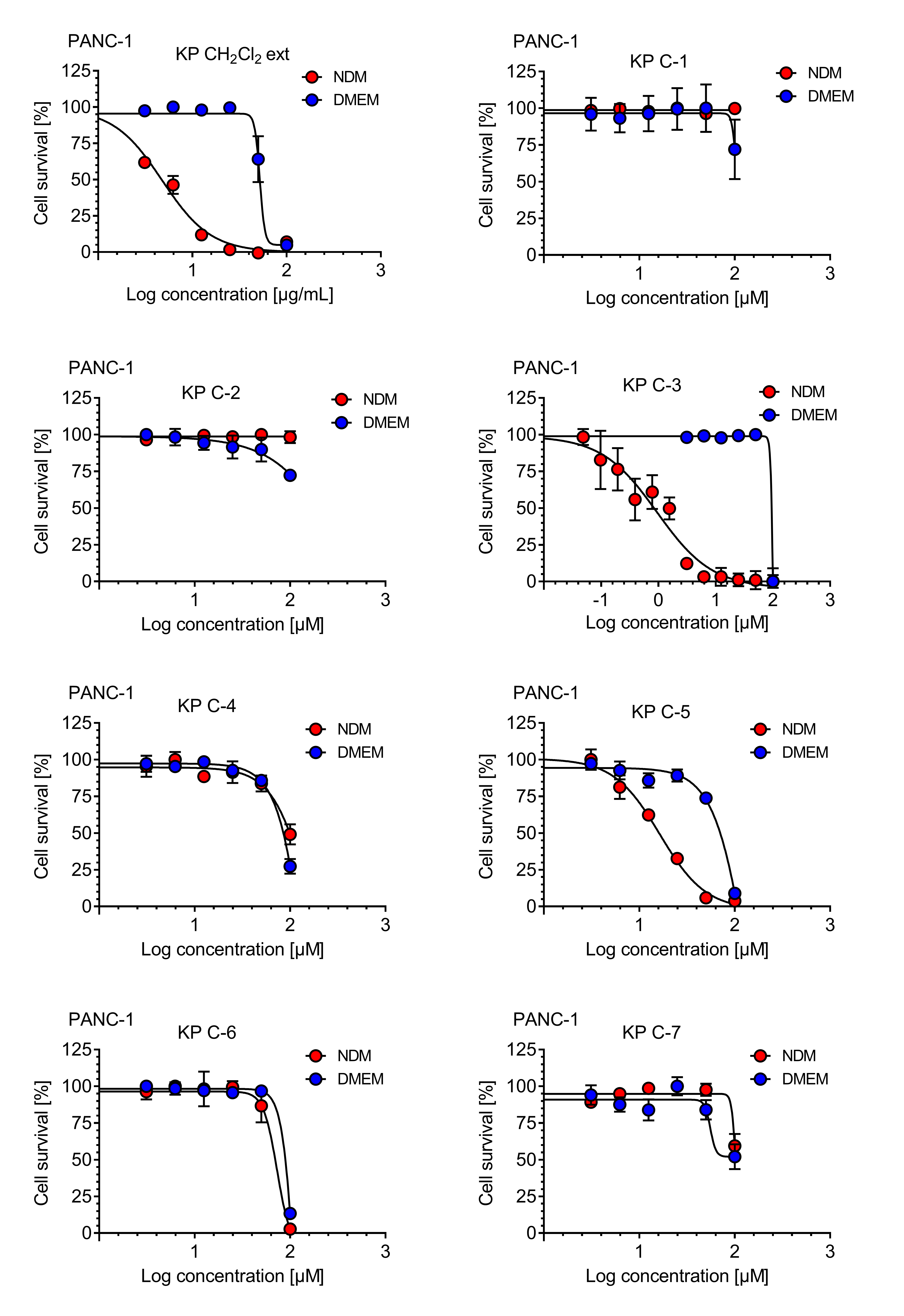


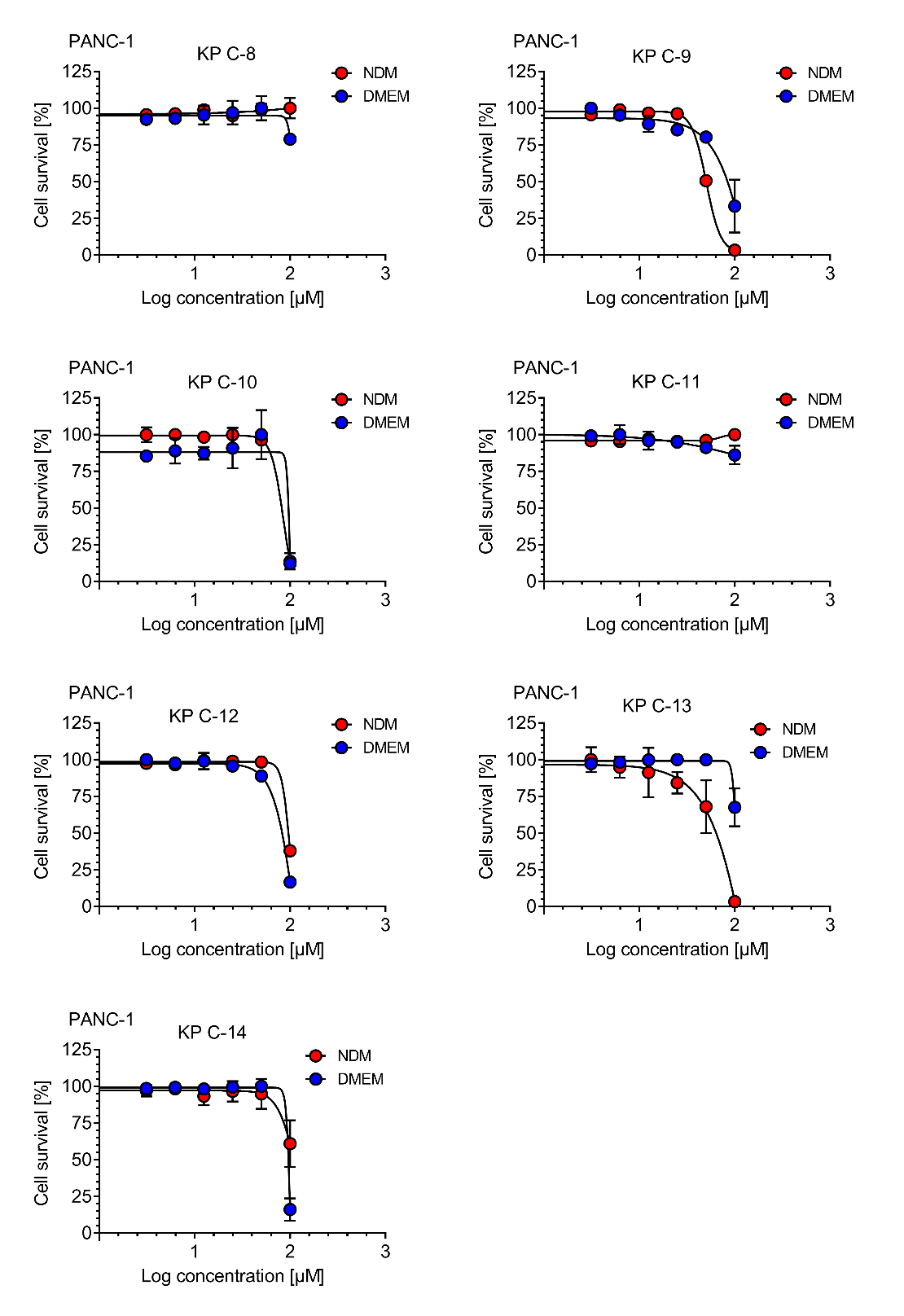

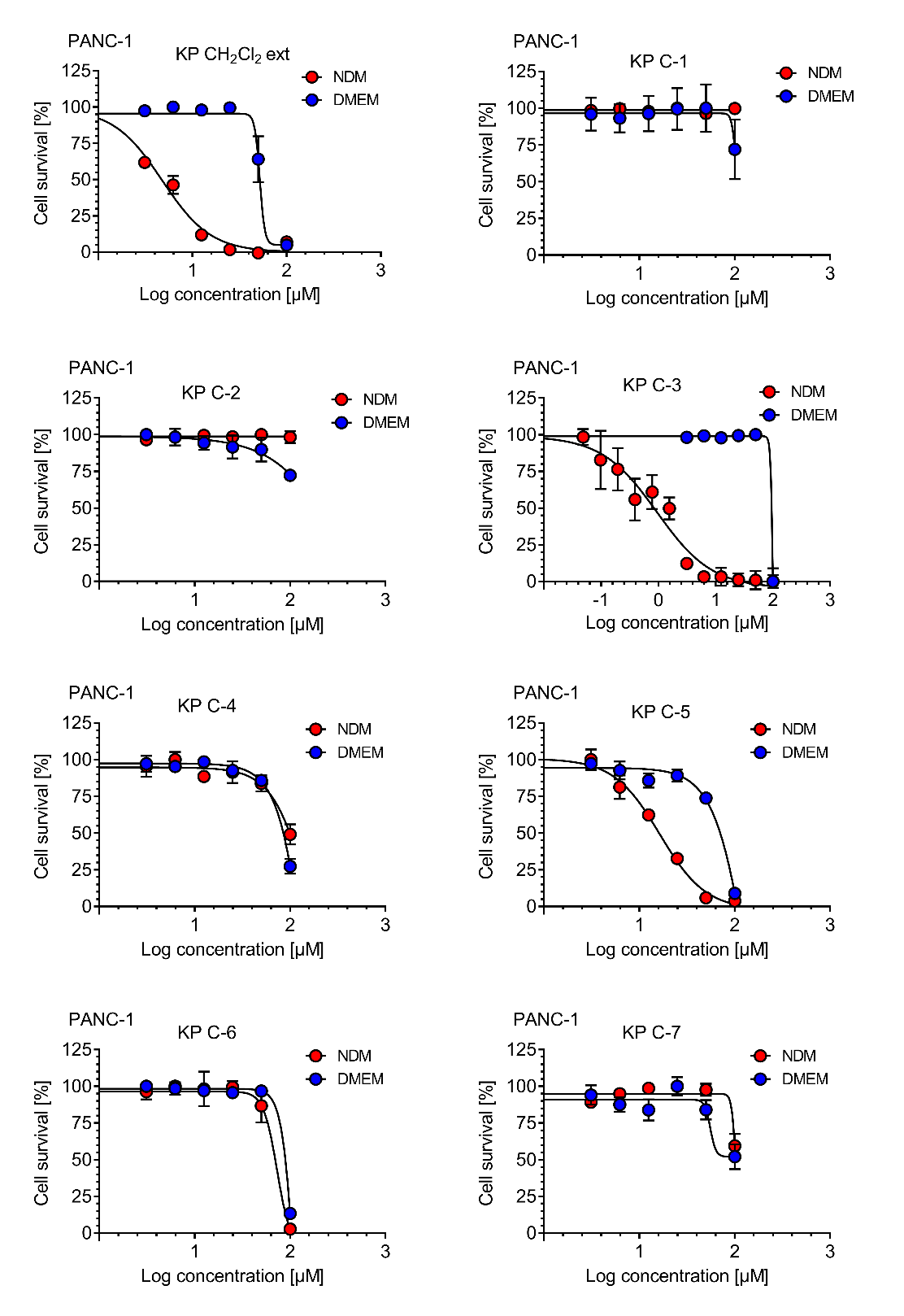

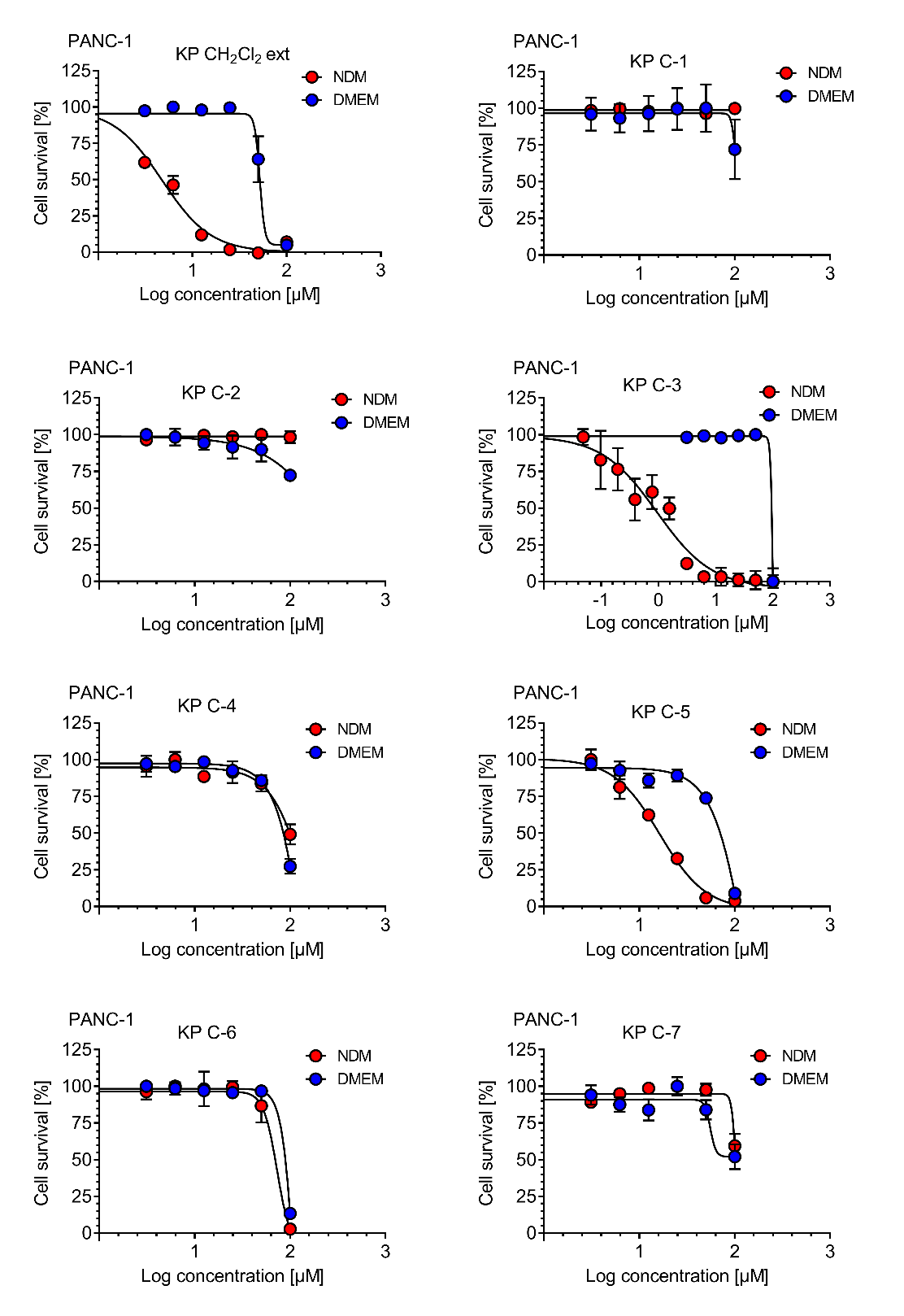

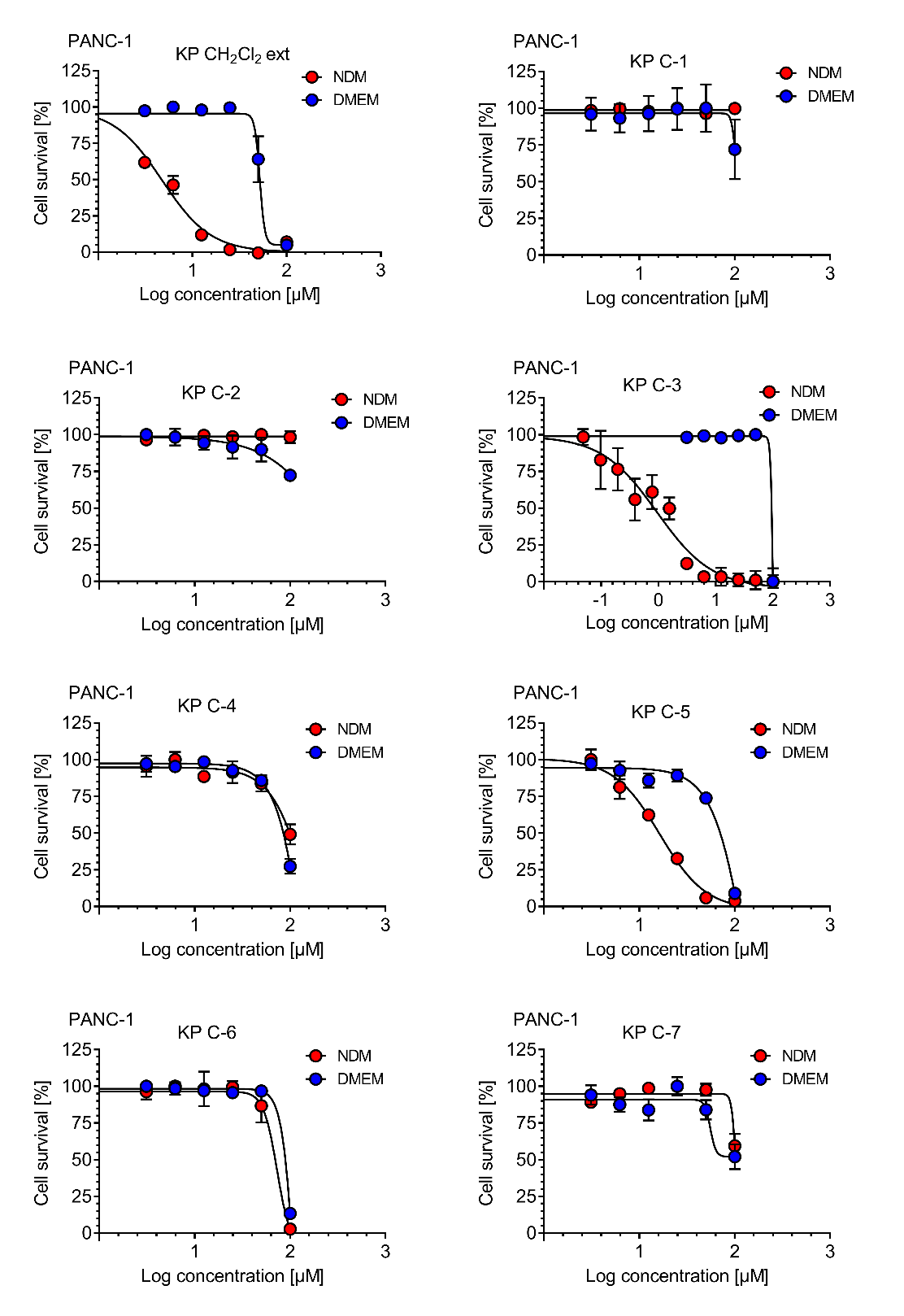

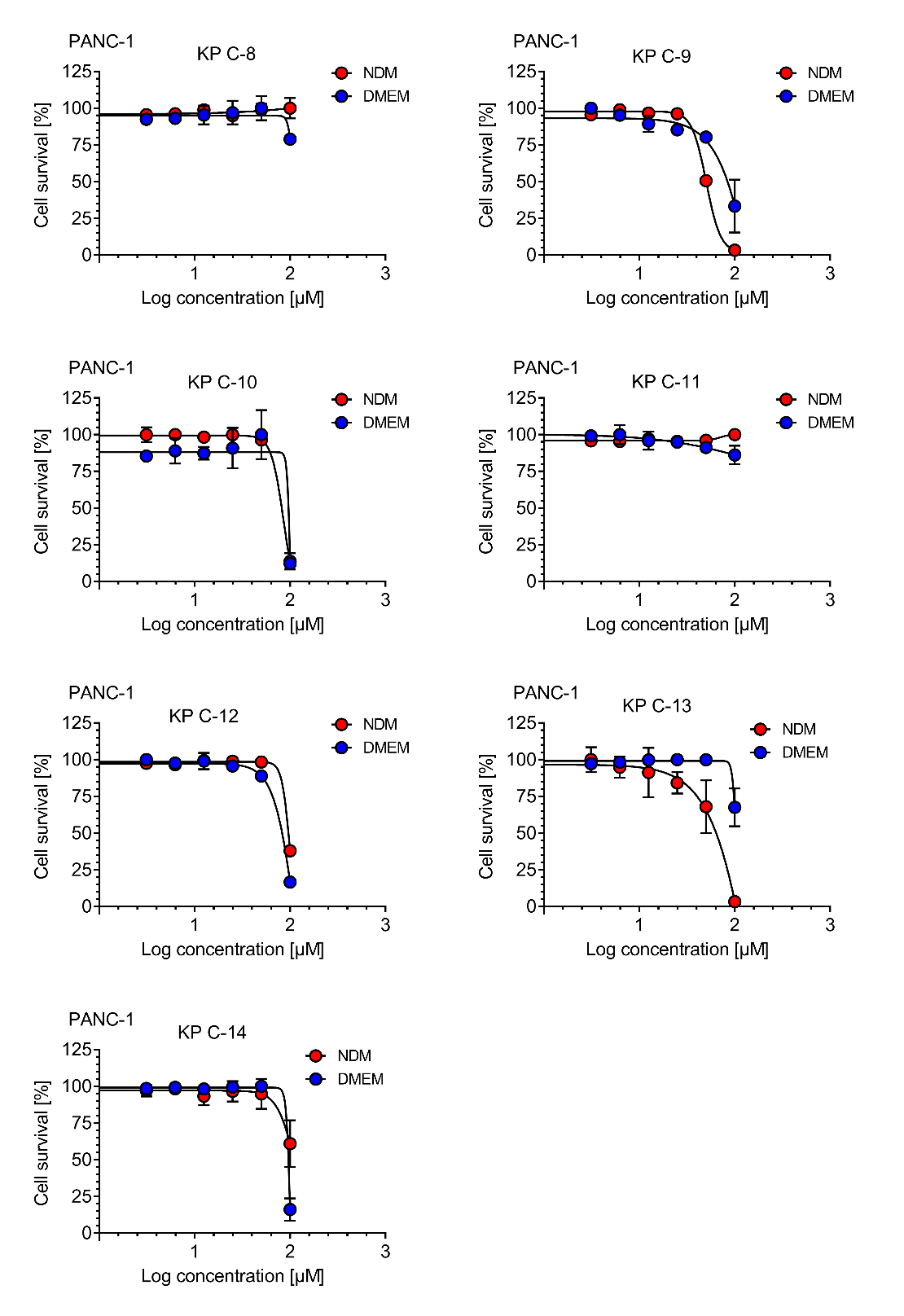

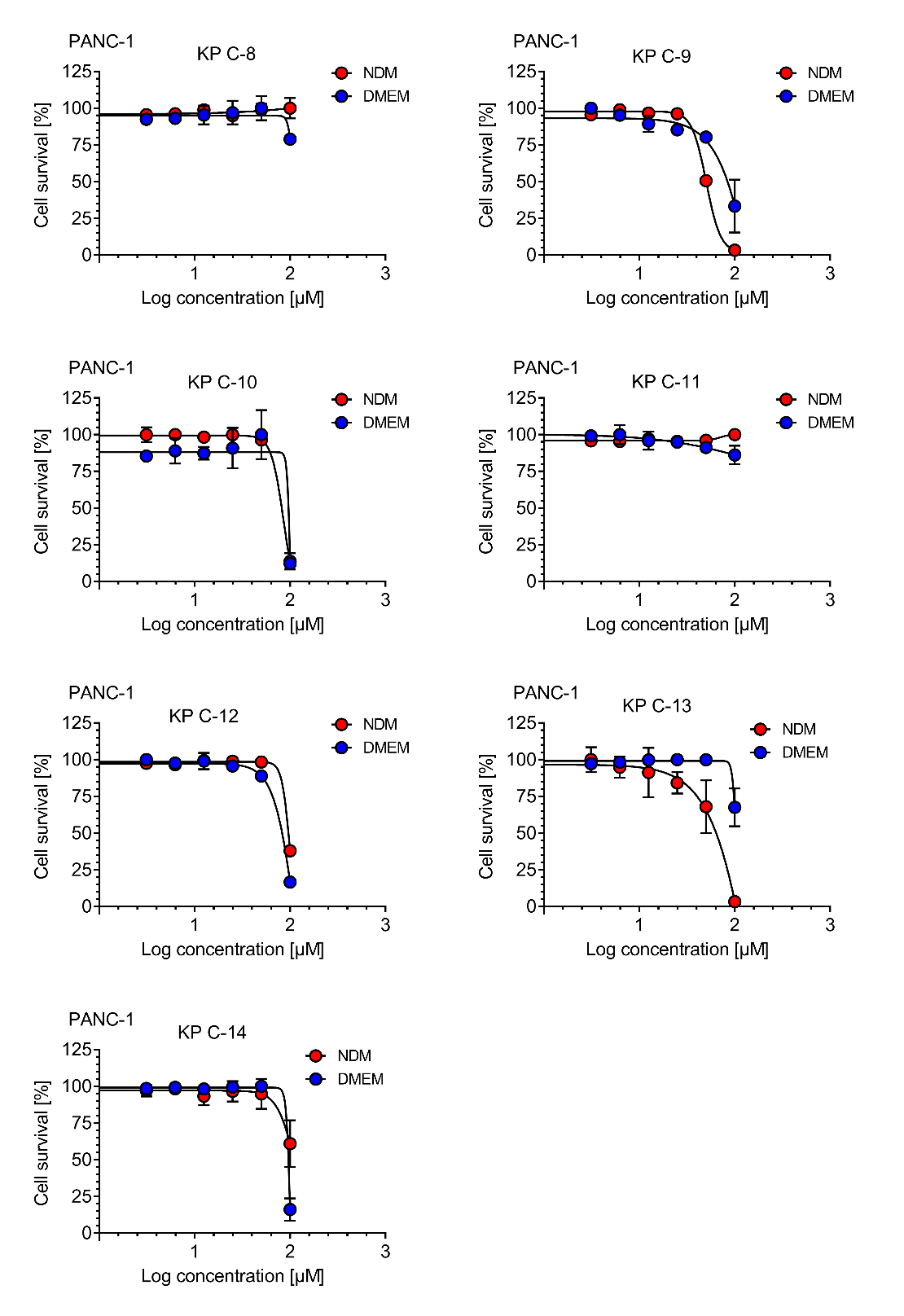

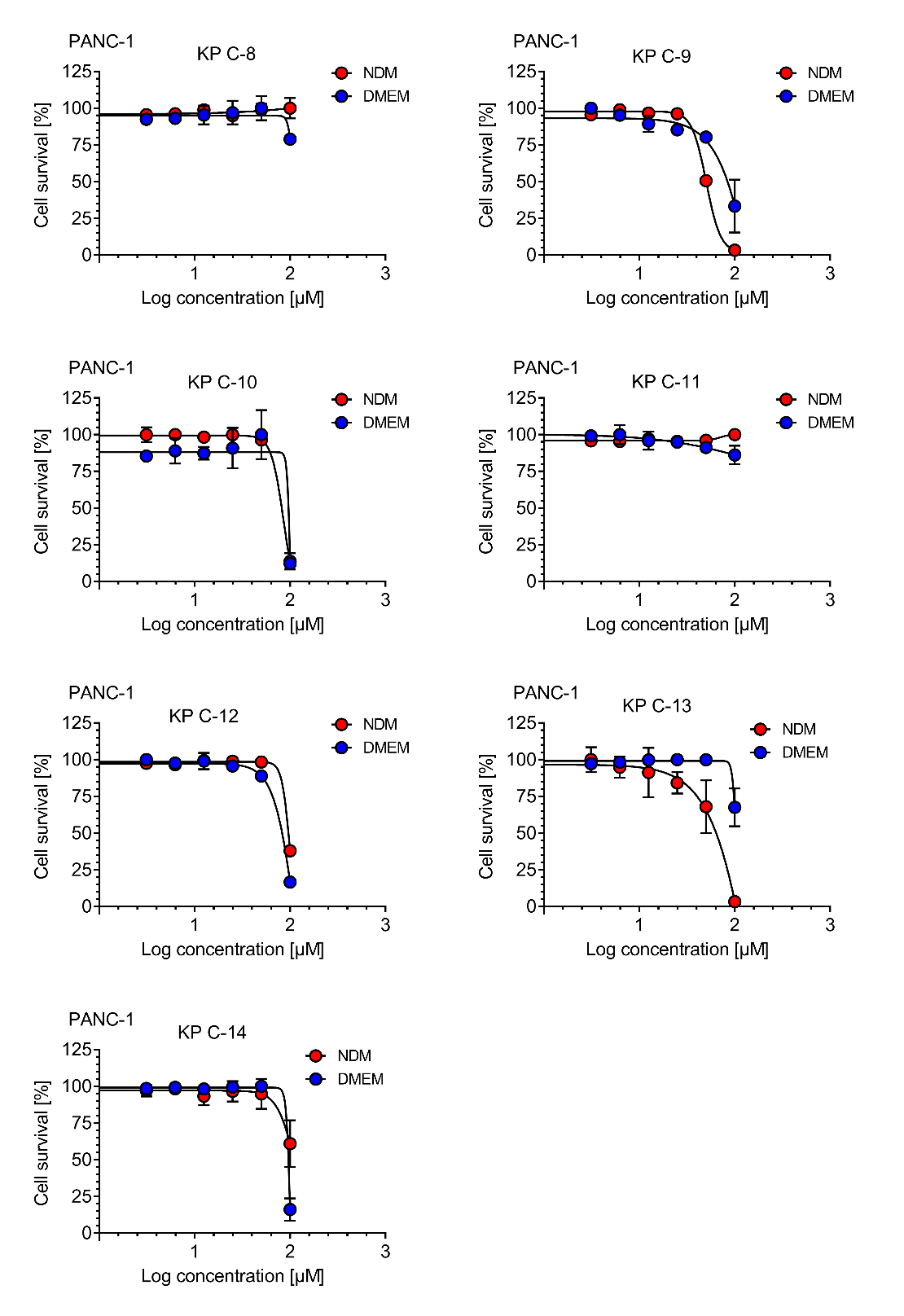


**Figure S9.** Preferential cytotoxic activity of *Kaempferia parviflora* CH_2_Cl_2_ extract and all isolated compounds against the PANC-1 in NDM and DMEM

**Figure S9.** Preferential cytotoxic activity of *Kaempferia parviflora* CH_2_Cl_2_ extract and all isolated compounds against the PANC-1 in NDM and DMEM


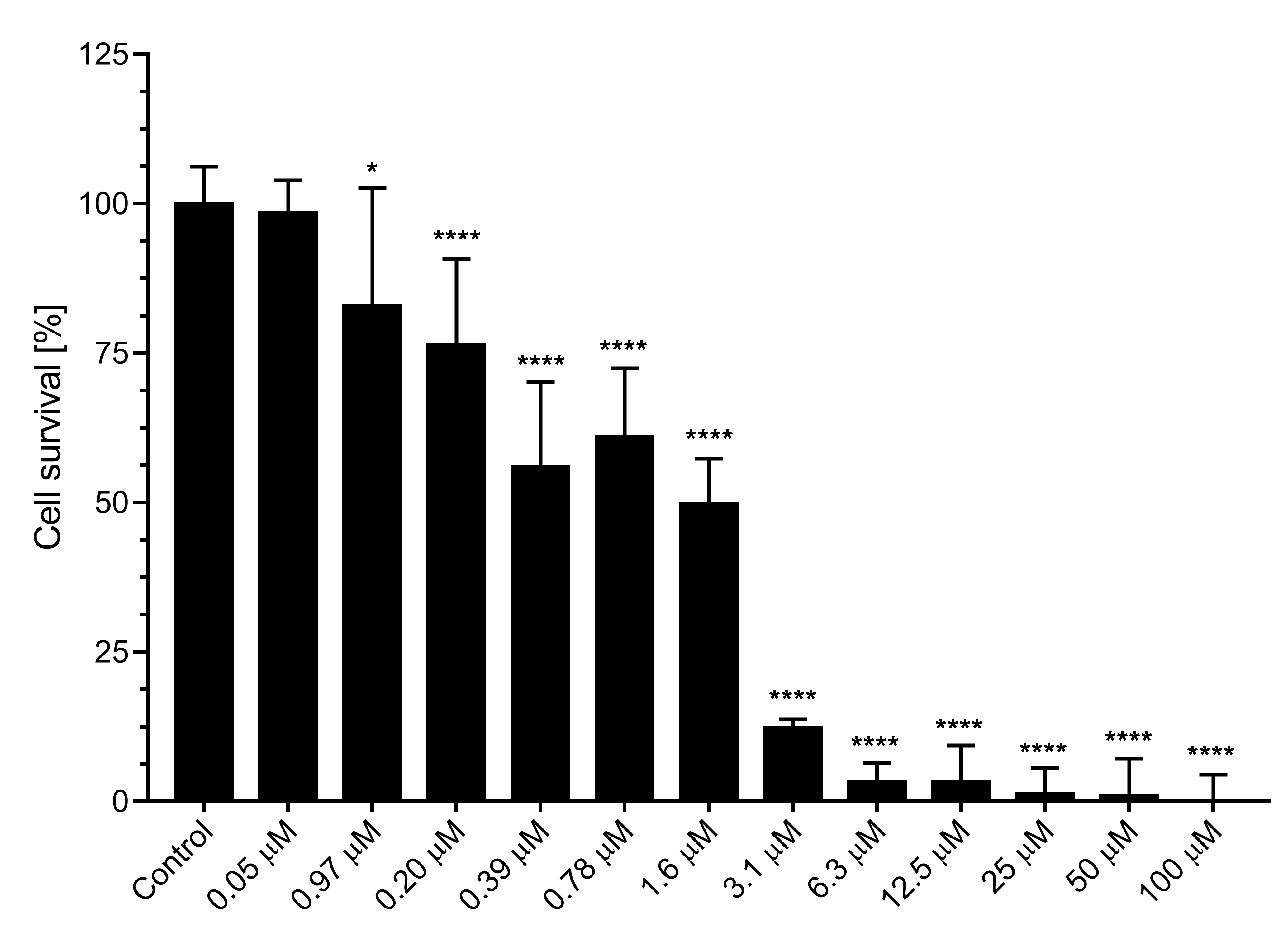


**Figure S10.** Preferential cytotoxic activity (anti-austerity activity) of 5-hydroxy-7-methoxyflavone (**3**) against the PANC-1 human pancreatic cancer cell line in nutrient-deprived medium (NDM). ****p < 0.0001, *p < 0.1 when compared with the untreated control group.
