# Supplementary material for: Anti-Austerity Activity of Thai Medicinal Plants: Chemical Constituents and Anti-Pancreatic Cancer Activities of Kaempferia parviflora
Source: Plants (Basel). 2021 Jan 25;10(2):229. doi: 10.3390/plants10020229 (PMC7911922; doi:10.3390/plants10020229)
Supplement: Supplementary file 1 [file plants-10-00229-s001.zip › Supplementary Material 2.docx]

*EXPERIMENTAL DETAILS*

A. Crystal Data

Empirical Formula C_16_H_16_O_7_

Formula Weight 320.30

Crystal Color, Habit colorless, plate

Crystal Dimensions 0.300 × 0.050 × 0.020 mm

Crystal System orthorhombic

Lattice Type Primitive

Lattice Parameters a = 12.9409(7) Å

b =  27.6849(15) Å

c =   4.3133(2) Å

V = 1545.32(14) Å^3^

Space Group P2_1_2_1_2 (#18)

Z value 4

D_calc_ 1.377 g/cm^3^

F_000_ 672.00

m(CuKα) 9.288 cm^-1^

B. Intensity Measurements

Diffractometer R-AXIS RAPID

Radiation CuKα (l = 1.54187 Å)

multi-layer mirror monochromated

Voltage, Current 40kV, 30mA

Temperature -100.0oC

Detector Aperture 460.0 × 256.0 mm

Data Images 225 exposures

w oscillation Range (c=54.0, f=0.0) 80.0 − 260.0°

Exposure Rate 70.0 sec./°

w oscillation Range (c=54.0, f=90.0) 80.0 − 260.0°

Exposure Rate 70.0 sec./°

w oscillation Range (c=54.0, f=180.0) 80.0 − 260.0°

Exposure Rate 70.0 sec./°

w oscillation Range (c=54.0, f=270.0) 80.0 − 260.0°

Exposure Rate 70.0 sec./°

w oscillation Range (c=0.0, f=0.0) 80.0 − 260.0°

Exposure Rate 70.0 sec./°

Detector Position 127.00 mm

Pixel Size 0.100 mm

2q_max_ 136.4°

No. of Reflections Measured Total: 16620

Unique: 2757 (R_int_ = 0.0831)

Parsons quotients (Flack x parameter): 375

Corrections Lorentz-polarization

Absorption

(trans. factors: 0.614 − 0.982)

C. Structure Solution and Refinement

Structure Solution Direct Methods (SHELXT Version 2018/2)

Refinement Full-matrix least-squares on F^2^

Function Minimized S w (Fo^2^ – Fc^2^)^2^

Least Squares Weights w = 1/ [ s^2^(Fo^2^) + (0.1782 . P)^2^

+ 0.0000 . P ]

where P = (Max(Fo^2^,0) + 2Fc^2^)/3

2qmax cutoff 136.4°

Anomalous Dispersion All non-hydrogen atoms

No. Observations (All reflections) 2757

No. Variables 210

Reflection/Parameter Ratio 13.13

Residuals: R1 (I>2.00s(I)) 0.0872

Residuals: R (All reflections) 0.1268

Residuals: wR2 (All reflections) 0.2796

Goodness of Fit Indicator 0.954

Flack parameter (Parsons' quotients = 375) 0.2(7)

Max Shift/Error in Final Cycle 0.000

Maximum peak in Final Diff. Map 0.34 e‑/Å^3^

Minimum peak in Final Diff. Map -0.30 e‑/Å^3^

Table 1. Atomic coordinates and B_iso_/B_eq_

atom    x    y    z  B_eq_

O1  0.5151(4)  0.5738(2)  0.8988(14)  5.10(13)

O2  0.7584(3)  0.57887(18)  0.8699(13)  4.79(12)

O3  0.7842(4)  0.67642(18)  0.7151(14)  4.98(12)

O4  0.5016(4)  0.67203(18)  0.7095(15)  5.10(13)

O5  0.5460(5)  0.7309(2)  1.0390(17)  7.20(18)

O6  0.7450(3)  0.48998(19)  0.4705(14)  4.99(12)

O7  0.6708(4)  0.4188(2)  0.5837(18)  7.01(17)

C1  0.5794(5)  0.5961(3)  0.675(2)  4.52(16)

C2  0.6776(5)  0.5666(3)  0.6506(19)  4.52(16)

C3  0.7730(5)  0.5898(3)  0.541(2)  4.68(16)

C4  0.7709(6)  0.6421(3)  0.463(2)  4.97(17)

C5  0.6839(6)  0.6719(3)  0.5743(19)  4.58(16)

C6  0.5999(5)  0.6486(3)  0.767(2)  4.50(16)

C7  0.6566(5)  0.5137(3)  0.612(2)  5.51(19)

C8  0.4837(7)  0.7131(3)  0.871(2)  5.22(18)

C9  0.3786(8)  0.7331(3)  0.788(3)  7.5(3)

C10  0.7399(6)  0.4416(3)  0.462(2)  5.4(2)

C11  0.8262(5)  0.4194(3)  0.291(2)  5.35(18)

C12  0.9040(6)  0.4471(3)  0.152(2)  5.7(2)

C13  0.9838(6)  0.4246(4)  0.000(3)  6.8(2)

C14  0.9836(7)  0.3753(5) -0.033(3)  7.8(3)

C15  0.9067(8)  0.3482(4)  0.095(3)  7.9(3)

C16  0.8268(7)  0.3703(3)  0.257(3)  7.0(2)

B_eq_ = 8/3 p2(U11(aa*)2 + U22(bb*)2 + U33(cc*)2 + 2U12(aa*bb*)cos g + 2U13(aa*cc*)cos b + 2U23(bb*cc*)cos a)

Table 2. Atomic coordinates and B_iso_ involving hydrogen atoms

atom    x    y    z  Biso

H1  0.54334  0.59560  0.46994  5.428

H1A  0.45360  0.57416  0.83639  6.125

H3  0.82289  0.56915  0.42360  5.611

H4  0.80144  0.65147  0.25812  5.966

H5  0.66191  0.69906  0.43641  5.492

H6  0.61775  0.65063  0.99181  5.400

H7A  0.64261  0.49899  0.81747  6.617

H7B  0.59480  0.50903  0.48066  6.617

H9A  0.38569  0.75618  0.61699  8.954

H9B  0.34907  0.74959  0.96883  8.954

H9C  0.33305  0.70666  0.72509  8.954

H12  0.90164  0.48140  0.16374  6.855

H13  1.03913  0.44319 -0.08239  8.157

H14  1.03744  0.35996 -0.14612  9.415

H15  0.90772  0.31400  0.07332  9.426

H16  0.77278  0.35133  0.34290  8.416

Table 3. Anisotropic displacement parameters

atom   U11   U22   U33   U12   U13   U23

O1 0.044(3) 0.076(4) 0.074(4)  0.001(3)  0.003(3)  0.001(3)

O2 0.044(3) 0.067(3) 0.072(4) -0.003(2) -0.003(3)  0.001(3)

O3 0.055(3) 0.068(3) 0.067(4) -0.010(2) -0.010(3)  -0.001(3)

O4 0.052(3) 0.059(3) 0.083(4)  0.008(2) -0.007(3)  -0.002(3)

O5 0.110(5) 0.062(4) 0.102(5)  0.009(3) -0.034(5) -0.012(4)

O6 0.046(3) 0.062(3) 0.082(4)  0.002(2)  0.010(3)  0.001(3)

O7 0.062(3) 0.068(3) 0.136(6) -0.003(3)  0.030(4)  -0.003(4)

C1 0.038(3) 0.065(5) 0.068(5) -0.005(3)  0.007(4) -0.009(4)

C2 0.038(3) 0.069(5) 0.064(5)  0.002(3)  -0.001(4)  0.002(4)

C3 0.048(4) 0.069(5) 0.061(6)  0.002(4)  0.004(4)  0.001(4)

C4 0.050(4) 0.070(5) 0.070(6) -0.013(4)  -0.003(4)  0.002(5)

C5 0.050(4) 0.065(4) 0.059(5)  -0.002(4) -0.009(4)  0.000(4)

C6 0.044(4) 0.065(4) 0.062(6)  -0.002(3) -0.006(4)  -0.000(4)

C7 0.043(4) 0.069(5) 0.097(7)  0.002(4)  0.010(4) -0.006(5)

C8 0.079(6) 0.054(5) 0.066(6)  0.008(4) -0.005(5)  0.003(5)

C9 0.095(7) 0.082(6) 0.106(8)  0.042(5) -0.011(7) -0.011(6)

C10 0.043(4) 0.069(5) 0.096(7) -0.004(4)  0.002(5) -0.008(5)

C11 0.044(4) 0.077(5) 0.083(6)  -0.001(4)  0.007(4)  -0.002(5)

C12 0.054(4) 0.081(5) 0.082(7)  -0.003(4)  0.016(5) -0.011(5)

C13 0.050(5) 0.106(8) 0.103(8)  0.001(5)  0.017(5) -0.019(7)

C14 0.052(5) 0.122(10) 0.124(10)  0.019(5)  0.007(6) -0.023(8)

C15 0.079(6) 0.087(7) 0.133(11)  0.026(6)  0.001(7) -0.021(7)

C16 0.066(5) 0.075(6) 0.125(9)  0.005(4)  0.016(6) -0.010(6)

The general temperature factor expression: exp(-2p2(a*2U11h2 + b*2U22k2 + c*2U33l2 + 2a*b*U12hk + 2a*c*U13hl + 2b*c*U23kl))

Table 4. Bond lengths (Å)

atom atom distance atom atom distance

O1 C1 1.416(10) O2 C2 1.451(9)

O2 C3 1.464(11) O3 C4 1.455(11)

O3 C5 1.438(9) O4 C6 1.449(9)

O4 C8 1.355(10) O5 C8 1.190(11)

O6 C7 1.453(9) O6 C10 1.342(10)

O7 C10 1.213(10) C1 C2 1.515(10)

C1 C6 1.530(11) C2 C3 1.471(10)

C2 C7 1.499(11) C3 C4 1.487(11)

C4 C5 1.477(11) C5 C6 1.512(11)

C8 C9 1.511(13) C10 C11 1.472(12)

C11 C12 1.400(12) C11 C16 1.369(13)

C12 C13 1.374(13) C13 C14 1.374(17)

C14 C15 1.364(16) C15 C16 1.389(15)

Table 5. Bond lengths involving hydrogens (Å)

atom atom distance atom atom distance

O1 H1A 0.840 C1 H1 1.000

C3 H3 1.000 C4 H4 1.000

C5 H5 1.000 C6 H6 1.000

C7 H7A 0.990 C7 H7B 0.990

C9 H9A 0.980 C9 H9B 0.980

C9 H9C 0.980 C12 H12 0.950

C13 H13 0.950 C14 H14 0.950

C15 H15 0.950 C16 H16 0.950

Table 6. Bond angles (o)

atom atom atom angle atom atom atom angle

C2 O2 C3 60.6(5) C4 O3 C5 61.4(5)

C6 O4 C8 116.0(6) C7 O6 C10 115.1(6)

O1 C1 C2 107.8(6) O1 C1 C6 109.9(7)

C2 C1 C6 112.7(6) O2 C2 C1 115.6(6)

O2 C2 C3 60.2(5) O2 C2 C7 115.6(6)

C1 C2 C3 119.3(6) C1 C2 C7 112.6(6)

C3 C2 C7 123.0(7) O2 C3 C2 59.2(5)

O2 C3 C4 114.8(7) C2 C3 C4 119.0(6)

O3 C4 C3 117.6(8) O3 C4 C5 58.7(5)

C3 C4 C5 118.9(7) O3 C5 C4 59.9(5)

O3 C5 C6 117.0(7) C4 C5 C6 119.3(7)

O4 C6 C1 103.2(5) O4 C6 C5 110.4(6)

C1 C6 C5 112.8(7) O6 C7 C2 110.2(6)

O4 C8 O5 123.0(8) O4 C8 C9 109.8(8)

O5 C8 C9 127.0(8) O6 C10 O7 122.9(8)

O6 C10 C11 113.1(7) O7 C10 C11 124.0(8)

C10 C11 C12 122.1(8) C10 C11 C16 118.2(8)

C12 C11 C16 119.6(8) C11 C12 C13 119.8(9)

C12 C13 C14 120.0(9) C13 C14 C15 120.5(10)

C14 C15 C16 120.2(11) C11 C16 C15 119.8(9)

Table 7. Bond angles involving hydrogens (o)

atom atom atom angle atom atom atom angle

C1 O1 H1A 109.5 O1 C1 H1 108.8

C2 C1 H1 108.8 C6 C1 H1 108.8

O2 C3 H3 117.0 C2 C3 H3 117.0

C4 C3 H3 117.0 O3 C4 H4 116.4

C3 C4 H4 116.4 C5 C4 H4 116.4

O3 C5 H5 116.3 C4 C5 H5 116.3

C6 C5 H5 116.2 O4 C6 H6 110.1

C1 C6 H6 110.1 C5 C6 H6 110.1

O6 C7 H7A 109.6 O6 C7 H7B 109.6

C2 C7 H7A 109.6 C2 C7 H7B 109.6

H7A C7 H7B 108.1 C8 C9 H9A 109.5

C8 C9 H9B 109.5 C8 C9 H9C 109.5

H9A C9 H9B 109.5 H9A C9 H9C 109.5

H9B C9 H9C 109.5 C11 C12 H12 120.1

C13 C12 H12 120.1 C12 C13 H13 120.0

C14 C13 H13 120.0 C13 C14 H14 119.8

C15 C14 H14 119.8 C14 C15 H15 119.9

C16 C15 H15 119.9 C11 C16 H16 120.1

C15 C16 H16 120.1

Table 8. Torsion Angles(o)

(Those having bond angles > 160 or < 20 degrees are excluded.)

atom1 atom2 atom3 atom4    angle atom1 atom2 atom3 atom4    angle

C2 O2 C3 C2 -0.0(3) C2 O2 C3 C4 110.2(6)

C3 O2 C2 C1 -110.6(7) C3 O2 C2 C3 -0.0(4)

C3 O2 C2 C7 114.9(7) C4 O3 C5 C4 -0.0(4)

C4 O3 C5 C6 109.9(7) C5 O3 C4 C3 -108.7(7)

C5 O3 C4 C5 0.0(4) C6 O4 C8 O5 3.8(12)

C6 O4 C8 C9 -179.8(6) C8 O4 C6 C1 156.4(6)

C8 O4 C6 C5 -82.8(8) C7 O6 C10 O7 -5.4(12)

C7 O6 C10 C11 174.7(6) C10 O6 C7 C2 171.8(7)

O1 C1 C2 O2 -86.6(7) O1 C1 C2 C3 -155.3(6)

O1 C1 C2 C7 49.2(8) O1 C1 C6 O4 -72.0(7)

O1 C1 C6 C5 168.9(5) C2 C1 C6 O4 167.9(6)

C2 C1 C6 C5 48.7(9) C6 C1 C2 O2 34.8(9)

C6 C1 C2 C3 -33.9(10) C6 C1 C2 C7 170.6(6)

O2 C2 C3 O2 0.0(2) O2 C2 C3 C4 -103.2(7)

O2 C2 C7 O6 -66.7(9) C1 C2 C3 O2 104.5(8)

C1 C2 C3 C4 1.3(11) C1 C2 C7 O6 157.4(7)

C3 C2 C7 O6 3.0(11) C7 C2 C3 O2 -102.8(8)

C7 C2 C3 C4 154.1(7) O2 C3 C4 O3 16.8(8)

O2 C3 C4 C5 -50.8(9) C2 C3 C4 O3 84.0(9)

C2 C3 C4 C5 16.3(12) O3 C4 C5 O3 0.0(3)

O3 C4 C5 C6 -106.0(7) C3 C4 C5 O3 106.5(8)

C3 C4 C5 C6 0.5(11) O3 C5 C6 O4 143.0(6)

O3 C5 C6 C1 -102.1(7) C4 C5 C6 O4 -148.0(7)

C4 C5 C6 C1 -33.2(10) O6 C10 C11 C12 -0.5(12)

O6 C10 C11 C16 -177.4(7) O7 C10 C11 C12 179.6(8)

O7 C10 C11 C16 2.7(14) C10 C11 C12 C13 179.0(7)

C10 C11 C16 C15 179.5(8) C12 C11 C16 C15 2.5(14)

C16 C11 C12 C13 -4.2(13) C11 C12 C13 C14 4.2(14)

C12 C13 C14 C15 -2.6(17) C13 C14 C15 C16 0.9(18)

C14 C15 C16 C11 -0.9(17)

Table 9. Possible hydrogen bonds

Donor  H Acceptor  D...A D-H H...A   D-H...A

 O1  H1A   O7^1^ 2.770(8) 0.84 1.95   163.80

Symmetry Operators:

(1) -X+1,-Y+1,Z

Table 10. Intramolecular contacts less than 3.60 Å

atom atom distance atom atom distance

O1 O2 3.154(6) O1 O4 2.844(8)

O1 C7 2.767(10) O2 O3 2.802(7)

O2 O6 3.009(8) O2 C5 3.031(9)

O2 C6 2.853(9) O3 C1 3.463(8)

O3 C2 3.351(9) O5 C5 3.142(10)

O5 C6 2.656(10) O6 C3 2.804(10)

O6 C12 2.743(10) O6 C16 3.600(11)

O7 C7 2.635(10) O7 C16 2.806(12)

C1 C4 2.934(10) C1 C8 3.571(11)

C2 C5 2.936(11) C3 C6 2.936(11)

C5 C8 3.107(12) C11 C14 2.757(14)

C12 C15 2.752(15) C13 C16 2.760(14)

Table 11. Intramolecular contacts less than 3.60 Å involving hydrogens

atom atom distance atom atom distance

O1 H6 2.539 O1 H7A 2.672

O1 H7B 2.745 O2 H1 3.307

O2 H4 3.363 O2 H6 2.745

O2 H7A 2.681 O2 H7B 3.323

O3 H3 3.264 O3 H6 2.564

O4 H1 2.416 O4 H1A 2.833

O4 H5 2.501 O4 H9A 2.799

O4 H9B 3.124 O4 H9C 2.383

O5 H5 3.128 O5 H6 2.418

O5 H9A 2.847 O5 H9B 2.618

O5 H9C 3.143 O6 H3 2.421

O6 H12 2.433 O7 H7A 2.465

O7 H7B 2.721 O7 H16 2.512

C1 H3 3.415 C1 H5 3.213

C1 H7A 2.877 C1 H7B 2.560

C2 H1A 3.015 C2 H4 3.310

C2 H6 2.860 C3 H1 2.992

C3 H5 3.379 C3 H6 3.264

C3 H7A 3.255 C3 H7B 3.223

C4 H1 3.215 C4 H6 3.033

C5 H1 2.824 C5 H3 3.428

C6 H1A 2.815 C6 H4 3.409

C7 H1 2.770 C7 H1A 3.262

C7 H3 2.766 C8 H5 2.998

C8 H6 2.505 C10 H7A 2.542

C10 H7B 2.649 C10 H12 2.693

C10 H16 2.586 C11 H13 3.259

C11 H15 3.243 C12 H14 3.235

C12 H16 3.255 C13 H15 3.233

C14 H12 3.237 C14 H16 3.243

C15 H13 3.232 C16 H12 3.250

C16 H14 3.245 H1 H1A 2.049

H1 H5 3.253 H1 H6 2.884

H1 H7A 3.324 H1 H7B 2.488

H1A H6 3.073 H1A H7A 3.213

H1A H7B 2.991 H3 H4 2.404

H3 H7A 3.479 H3 H7B 3.398

Table 11. Intramolecular contacts less than 3.60 Å involving hydrogens (continued)

atom atom distance atom atom distance

H3 H12 2.863 H4 H5 2.364

H5 H6 2.804 H12 H13 2.326

H13 H14 2.321 H14 H15 2.309

H15 H16 2.339

Table 12. Intermolecular contacts less than 3.60 Å

atom atom distance atom atom distance

O1 O7^1^ 2.770(8) O1 C1^2^ 3.505(11)

O1 C7^1^ 3.511(9) O2 O6^2^ 3.577(8)

O2 C2^2^ 3.543(10) O2 C3^2^ 2.915(11)

O2 C4^2^ 3.103(11) O2 C13^3^ 3.384(9)

O2 C14^3^ 3.596(11) O3 C4^2^ 3.365(12)

O3 C9^4^ 3.532(12) O3 C9^5^ 3.514(12)

O3 C14^3^ 3.501(11) O4 O5^6^ 3.369(9)

O4 O7^1^ 3.405(7) O5 O4^2^ 3.369(9)

O5 C5^2^ 3.344(10) O5 C15^7^ 3.353(13)

O6 O2^6^ 3.577(8) O7 O1^1^ 2.770(8)

O7 O4^1^ 3.405(7) O7 C1^1^ 3.288(9)

C1 O1^6^ 3.505(11) C1 O7^1^ 3.288(9)

C2 O2^6^ 3.543(10) C3 O2^6^ 2.915(11)

C4 O2^6^ 3.103(11) C4 O3^6^ 3.365(12)

C5 O5^6^ 3.344(10) C7 O1^1^ 3.511(9)

C9 O3^8^ 3.532(12) C9 O3^9^ 3.514(12)

C13 O2^10^ 3.384(9) C14 O2^10^ 3.596(11)

C14 O3^10^ 3.501(11) C15 O5^11^ 3.353(13)

Symmetry Operators:

(1) -X+1,-Y+1,Z (2) X,Y,Z+1

(3) -X+2,-Y+1,Z+1 (4) X+1/2,-Y+1/2+1,-Z+1

(5) X+1/2,-Y+1/2+1,-Z+2 (6) X,Y,Z-1

(7) -X+1/2+1,Y+1/2,-Z+1 (8) X+1/2-1,-Y+1/2+1,-Z+1

(9) X+1/2-1,-Y+1/2+1,-Z+2 (10) -X+2,-Y+1,Z-1

(11) -X+1/2+1,Y+1/2-1,-Z+1

Table 13. Intermolecular contacts less than 3.60 Å involving hydrogens

atom atom distance atom atom distance

O1 H1^1^ 2.562 O1 H7A^2^ 2.890

O1 H7B^1^ 3.253 O1 H7B^2^ 3.246

O2 H3^1^ 2.544 O2 H4^1^ 2.675

O2 H12^1^ 3.511 O2 H13^3^ 2.698

O2 H14^3^ 3.139 O3 H4^1^ 2.452

O3 H5^1^ 3.546 O3 H9A^4^ 2.694

O3 H9B^5^ 2.600 O3 H14^3^ 2.589

O4 H6^6^ 3.492 O5 H5^1^ 2.443

O5 H9A^1^ 3.318 O5 H15^7^ 2.426

O6 H1A^2^ 3.500 O6 H7A^6^ 3.123

O7 H1^2^ 2.842 O7 H1A^2^ 1.954

O7 H9C^2^ 3.527 C1 H1^1^ 3.461

C1 H6^6^ 3.348 C3 H4^1^ 3.553

C3 H6^6^ 3.532 C3 H13^3^ 3.064

C3 H14^3^ 3.127 C4 H4^1^ 3.464

C4 H6^6^ 2.847 C4 H9A^4^ 3.201

C4 H14^3^ 3.001 C5 H4^1^ 3.367

C5 H6^6^ 2.719 C5 H9A^4^ 3.386

C6 H1^1^ 3.449 C6 H4^1^ 3.362

C6 H5^1^ 3.307 C6 H6^6^ 3.350

C7 H1A^2^ 2.980 C7 H7A^6^ 3.458

C7 H7B^2^ 3.362 C8 H5^1^ 3.378

C8 H15^7^ 3.135 C9 H4^8^ 3.353

C9 H5^8^ 3.511 C9 H9B^6^ 3.584

C10 H1A^2^ 3.011 C10 H7A^6^ 3.440

C12 H12^9^ 3.200 C12 H13^9^ 3.284

C13 H3^10^ 3.531 C13 H3^9^ 3.102

C13 H12^9^ 3.076 C14 H3^9^ 3.538

C14 H4^9^ 3.141 C16 H9C^2^ 3.592

H1 O1^6^ 2.562 H1 O7^2^ 2.842

H1 C1^6^ 3.461 H1 C6^6^ 3.449

H1 H1A^6^ 3.028 H1 H6^6^ 2.739

H1 H7B^2^ 3.404 H1A O6^2^ 3.500

H1A O7^2^ 1.954 H1A C7^2^ 2.980

H1A C10^2^ 3.011 H1A H1^1^ 3.028

H1A H7A^2^ 2.379 H1A H7B^2^ 2.837

H3 O2^6^ 2.544 H3 C13^9^ 3.102

Table 13. Intermolecular contacts less than 3.60 Å involving hydrogens (continued)

atom atom distance atom atom distance

H3 C13^3^ 3.531 H3 C14^9^ 3.538

H3 H13^9^ 2.840 H3 H13^3^ 2.801

H3 H14^3^ 3.250 H4 O2^6^ 2.675

H4 O3^6^ 2.452 H4 C3^6^ 3.553

H4 C4^6^ 3.464 H4 C5^6^ 3.367

H4 C6^6^ 3.362 H4 C9^4^ 3.353

H4 C14^9^ 3.141 H4 H6^6^ 2.640

H4 H9A^4^ 2.831 H4 H9B^4^ 2.973

H4 H14^9^ 2.736 H4 H14^3^ 3.324

H5 O3^6^ 3.546 H5 O5^6^ 2.443

H5 C6^6^ 3.307 H5 C8^6^ 3.378

H5 C9^4^ 3.511 H5 H6^6^ 2.409

H5 H9A^4^ 3.158 H5 H9B^4^ 3.308

H5 H9C^4^ 3.493 H6 O4^1^ 3.492

H6 C1^1^ 3.348 H6 C3^1^ 3.532

H6 C4^1^ 2.847 H6 C5^1^ 2.719

H6 C6^1^ 3.350 H6 H1^1^ 2.739

H6 H4^1^ 2.640 H6 H5^1^ 2.409

H7A O1^2^ 2.890 H7A O6^1^ 3.123

H7A C7^1^ 3.458 H7A C10^1^ 3.440

H7A H1A^2^ 2.379 H7A H7B^1^ 2.940

H7A H7B^2^ 3.406 H7B O1^6^ 3.253

H7B O1^2^ 3.246 H7B C7^2^ 3.362

H7B H1^2^ 3.404 H7B H1A^2^ 2.837

H7B H7A^6^ 2.940 H7B H7A^2^ 3.406

H7B H7B^2^ 2.504 H9A O3^8^ 2.694

H9A O5^6^ 3.318 H9A C4^8^ 3.201

H9A C5^8^ 3.386 H9A H4^8^ 2.831

H9A H5^8^ 3.158 H9A H9B^6^ 2.841

H9A H15^7^ 3.390 H9B O3^11^ 2.600

H9B C9^1^ 3.584 H9B H4^8^ 2.973

H9B H5^8^ 3.308 H9B H9A^1^ 2.841

H9B H9C^1^ 3.478 H9B H14^7^ 3.475

H9B H16^12^ 3.591 H9C O7^2^ 3.527

H9C C16^2^ 3.592 H9C H5^8^ 3.493

H9C H9B^6^ 3.478 H9C H15^12^ 3.506

H9C H16^2^ 2.678 H9C H16^12^ 3.399

Table 13. Intermolecular contacts less than 3.60 Å involving hydrogens (continued)

atom atom distance atom atom distance

H12 O2^6^ 3.511 H12 C12^9^ 3.200

H12 C13^9^ 3.076 H12 H12^9^ 2.746

H12 H13^9^ 2.464 H13 O2^10^ 2.698

H13 C3^10^ 3.064 H13 C12^9^ 3.284

H13 H3^10^ 2.801 H13 H3^9^ 2.840

H13 H12^9^ 2.464 H13 H13^9^ 3.305

H14 O2^10^ 3.139 H14 O3^10^ 2.589

H14 C3^10^ 3.127 H14 C4^10^ 3.001

H14 H3^10^ 3.250 H14 H4^10^ 3.324

H14 H4^9^ 2.736 H14 H9B^13^ 3.475

H15 O5^13^ 2.426 H15 C8^13^ 3.135

H15 H9A^13^ 3.390 H15 H9C^14^ 3.506

H16 H9B^14^ 3.591 H16 H9C^14^ 3.399

H16 H9C^2^ 2.678

Symmetry Operators:

(1) X,Y,Z+1 (2) -X+1,-Y+1,Z

(3) -X+2,-Y+1,Z+1 (4) X+1/2,-Y+1/2+1,-Z+1

(5) X+1/2,-Y+1/2+1,-Z+2 (6) X,Y,Z-1

(7) -X+1/2+1,Y+1/2,-Z+1 (8) X+1/2-1,-Y+1/2+1,-Z+1

(9) -X+2,-Y+1,Z (10) -X+2,-Y+1,Z-1

(11) X+1/2-1,-Y+1/2+1,-Z+2 (12) -X+1,-Y+1,Z+1

(13) -X+1/2+1,Y+1/2-1,-Z+1 (14) -X+1,-Y+1,Z-1
